# A Network Analysis of the Human T-Cell Activation Gene Network Identifies Jagged1 as a Therapeutic Target for Autoimmune Diseases

**DOI:** 10.1371/journal.pone.0001222

**Published:** 2007-11-21

**Authors:** Ricardo Palacios, Joaquin Goni, Ivan Martinez-Forero, Jaime Iranzo, Jorge Sepulcre, Ignacio Melero, Pablo Villoslada

**Affiliations:** 1 Department of Neurology, Clinica Universitaria de Navarra and Center for Applied Medical Research, University of Navarra, Pamplona, Spain; 2 Department of Physics and Applied Mathematics, University of Navarra, Pamplona, Spain; 3 Hepathology and Gene Therapy, Center for Applied Medical Research, University of Navarra, Pamplona, Spain; Centre de Recherche Public-Santé, Luxembourg

## Abstract

Understanding complex diseases will benefit the recognition of the properties of the gene networks that control biological functions. Here, we set out to model the gene network that controls T-cell activation in humans, which is critical for the development of autoimmune diseases such as Multiple Sclerosis (MS). The network was established on the basis of the quantitative expression from 104 individuals of 20 genes of the immune system, as well as on biological information from the Ingenuity database and Bayesian inference. Of the 31 links (gene interactions) identified in the network, 18 were identified in the Ingenuity database and 13 were new and we validated 7 of 8 interactions experimentally. In the MS patients network, we found an increase in the weight of gene interactions related to Th1 function and a decrease in those related to Treg and Th2 function. Indeed, we found that IFN-ß therapy induces changes in gene interactions related to T cell proliferation and adhesion, although these gene interactions were not restored to levels similar to controls. Finally, we identify JAG1 as a new therapeutic target whose differential behaviour in the MS network was not modified by immunomodulatory therapy. In vitro treatment with a Jagged1 agonist peptide modulated the T-cell activation network in PBMCs from patients with MS. Moreover, treatment of mice with experimental autoimmune encephalomyelitis with the Jagged1 agonist ameliorated the disease course, and modulated Th2, Th1 and Treg function. This study illustrates how network analysis can predict therapeutic targets for immune intervention and identified the immunomodulatory properties of Jagged1 making it a new therapeutic target for MS and other autoimmune diseases.

## Introduction

Understanding the structure and dynamics of biological networks may prove critical to unravel complex traits and diseases, such as autoimmune diseases [1]. In the immune response, T cells interact with antigen-presenting cells in a complex process that generates changes in gene expression. These changes underlie cell differentiation, and effector and regulatory events, as well as promoting the acquisition of a panel of adhesion molecules that guide cells to the appropriate tissues [2], [3]. Several evidences indicates gene deregulation within the immune system in autoimmune diseases [4], [5], such as in Multiple Sclerosis (MS) [6]. Several studies suggest that T-cell activation and the ensuing differentiation to effector cells or is one of the most critical process in controlling autoimmunity, as well as maintaining the balance between effector and regulatory mechanisms [7]–[11]. However, despite the many molecular and cellular studies, we still lack a comprehensive understanding of how the immune system is controlled and how autoimmune diseases arise. Given the complex interactions between the cells and molecules that regulate this process, a systems approach to analyse these processes might identify critical functional interactions that are disturbed in autoimmune diseases. Moreover, the identification of such pathological interactions might facilitate the development of new therapeutic targets [12], [13].

MS is a chronic inflammatory and neurodegenerative disease of the central nervous system [14]. MS is characterized by the presence of plaques composed by chronic inflammatory infiltrates, including T and B cells as well as monocytes into the brain, accompanied by the presence of large areas of demyelination and axonal loss [6]. MS is the second cause of permanent disability in young adults after spinal cord injury and due to its chronic nature imposes a significant health and social cost in western countries. Although current immunotherapies are able to modify disease course, we still need to develop more effective and safe therapies for improving the quality of life of patients.

The development of network theory is providing important insights into gene and protein networks [15] . However, the translation of such advances to humans complex diseases such as autoimmune diseases is confronted with many challenges, such as incomplete knowledge of the molecules involved, lack of quantitative data, the higher degree of complexity and the limited availability of analytical methods. Among several methods of network analysis for reconstructing network topology from experimental datasets [16], Bayesian networks are those that offer the best results [17], [18]. In human complex diseases, the use of different clinical phenotypes such as quantitative traits, disease subtypes or therapies, can introduce meaningful perturbations into a network to help infer its topology [19].

The aim of our study was to assess the functional properties of the gene network that controls the T-cell activation processes in healthy circumstances and in an autoimmune disease such as MS. Furthermore, we assessed the effect of immunotherapy in such a gene network. In addition we were interested in identifying new therapeutic targets at the systems level. In order to achieve this objective, we performed a network analysis using quantitative measurements of gene expression obtained by real time PCR from a small number of well-known genes involved in T-cell activation, as well as using prior biological information. We limited our study to a set of 20 genes for two reasons: 1) we were interested in obtaining a balanced matrix considering number of genes, subjects and perturbations; and 2) the limited amount of RNA from every individual in which assess the gene expression levels by real time PCR in the same sample. We employed a Bayesian approach to obtain an accurate reconstruction of the network and indeed, we assessed the qualitative and quantitative network properties using several new methods from systems biology. This systems approach to autoimmunity revealed functional differences in the gene network that controls T-cell activation that cannot be captured with previous methods. Moreover, we show how this approach can be useful in translational clinical research by evaluating the effect of current therapies, and by identifying new therapeutic targets for immunotherapy, such as the Jagged1-Notch pathway.

## Results

### Network analysis of the T-cell activation gene network

**Figure 1:**
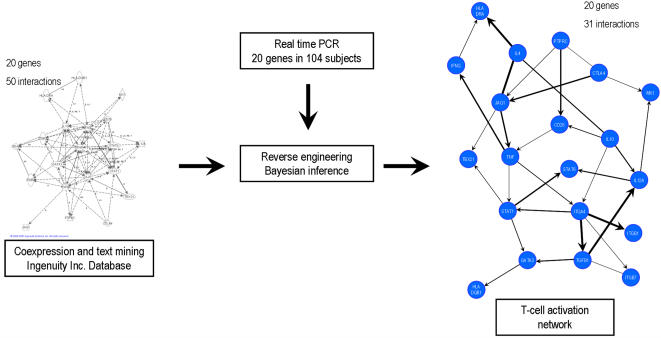
Network analysis of the human T-cell activation network: The structural network was obtained from co-expression analysis using the Ingenuity database. The structural network has 20 genes and we identified 50 links. Using the structural network as a template and the experimental dataset (gene expression levels of the 20 genes from 104 subjects quantified by real time-PCR), we reconstructed the T-cell activation network. The network contains the 20 genes and we identified 31 links (see Table 1 for information about the weight, direction and previous biological knowledge of the links).

In order to obtain a more accurate network, we decided to focus on a small set of genes for which the biological information was more complete, they were previously implicated in MS and having them distributed among five basic functions associated with T-cell activation. In this way, we were able to obtain quantitative data and a good balance between the size of the network and the number of possible perturbations related to the different disease phenotypes and therapies [17]. Thus, the experimental dataset was obtained by assessing the gene expression levels of the 20 genes by real time PCR in a cohort of 104 subjects, composed by healthy controls and patients with MS (see Table 1 for details of the clinical characteristics and Table S1 for statistically summary of gene expression levels). Our approach profits the presence of different phenotypes (healthy, patient condition or different immunotherapies) as perturbations to the system in order to improve the ability of Bayesian inference to identify the right interactions. In the other hand, since Bayesian algorithms might lead to several different results using the same experimental dataset, we also feed the algorithm with a structural network template to decrease the number of different outputs and to increase its accuracy. We obtained the template (*structural network*) by applying biological knowledge from co-expression studies available at the Ingenuity database (http://www.ingenuity.com/). The resulting *structural network* identified 50 links (Fig 1). Using this *structural network* as a template, we inferred the topology of the human T-cell activation network by using the experimental dataset performing a Bayesian network approach (see methods).

**Table 1:** Demographic and clinical data of patients and controls

	HC	MS
N =	52	52
Male/Female	26/26	26/26
Age (years)	45.6±17.7	39.3±10.4
EDSS score	–	3.00±1.82
MSFC score	–	−0.02±0.66
Disease duration (years)	–	6.70±6.45
Immunomodulatory therapy (yes/no)	–	25/27

The inferred network model (Fig 1 and Table 2) has 31 links between the 20 genes, of which 26 were arcs and 4 were edges. We found that hubs in the T-cell activation network differ from those in the *structural* network, indicating that the experimental dataset was informative to overcome the literature bias by which genes studied for many years (e.g. IFNγ) are cited more often than new genes (e.g. transcription factors). Moreover, because cycles are not permitted in Bayesian networks, the links identified represent a selection of the most relevant interactions in the network, including the removal of the redundant ones such as autocrine loops. Of the 31 links identified, 18 were identified in the gene co-expression searches in the Ingenuity database, or were described in the biological literature (supplementary Table S2 and S3) and as such, they were already present in the *structural* network. The remaining 32 links present in the structural network and absent in the final network were discarded by the algorithm for the following reasons: 1) the gene interaction is not functional in the tissue studied (PBMC); 2) the magnitude of the interaction was not enough for being detected based in the gene co-expression levels; 3) removal of cycles imposed by the Bayesian inference. For 15 of the 18 predicted links, the direction of the interaction was correctly inferred, while for the other three interactions there was insufficient information for the Bayesian algorithm to define this parameter. The Bayesian algorithm found 13 new links that have not been previously reported or identified by bioinformatics analyses of the gene co-expression database. Due to the small size of our network, we did not calculate common network parameters such as degree, mean path-length or clustering coefficient.

**Table 2:** Gene interactions identified in the T-cell activation network.

Parent	Child	Type	Relative weight	Category	Orientation
TGFB1	IL12A	Arc	1.00	New	–
ITGA4	TGFB1	Arc	0.99	Predicted	Reverse
JAG1	IL4	Edge	0.96	Predicted	Right
ITGA4	ITGB1	Arc	0.86	Predicted	Right
IL4	HLA-DRA	Arc	0.78	New	–
PTPRC	CD28	Arc	0.76	Predicted	Reverse
TNF	IFNG	Arc	0.73	New	–
JAG1	TNF	Arc	0.68	New	–
CTLA4	JAG1	Arc	0.65	New	–
STAT1	STAT6	Arc	0.64	Predicted	Right
TGFB1	GATA3	Arc	0.61	Predicted	Right
IL10	IL12A	Arc	0.56	New	–
IL12A	STAT6	Arc	0.54	New	–
ITGA4	STAT1	Arc	0.51	New	–
IL4	IL10	Edge	0.49	Predicted	Right
STAT1	GATA3	Arc	0.48	Predicted	Right
IL12A	MX1	Arc	0.46	New	–
IL10	CD28	Arc	0.45	Predicted	Reverse
TNF	ITGA4	Arc	0.43	Predicted	Right
GATA3	HLA-DQB1	Arc	0.40	New	–
STAT1	TBX21	Arc	0.38	Predicted	Right
PTPRC	JAG1	Arc	0.37	New	–
TGFB1	ITGB7	Edge	0.37	Predicted	Right
IFNG	HLA-DRA	Arc	0.36	Predicted	Right
PTPRC	CTLA4	Edge	0.35	Predicted	Right
ITGA4	ITGB7	Arc	0.33	Predicted	Right
TNF	STAT1	Arc	0.33	New	–
CTLA4	MX1	Arc	0.31	New	–
CD28	TNF	Arc	0.30	Predicted	Right
IL10	ITGA4	Arc	0.30	Predicted	Right
JAG1	TBX21	Arc	0.26	Predicted	Right

Links whose direction cannot be changed without changing the probabilistic relations encoded are named arcs, otherwise they are edges. If there is an arc from gene A to another gene B, then we say that A is a parent of B and B is a child of A. The relative weight was measured with the Kullback-Leibler (KL) divergence indicating the contribution of each link with respect to the complete network structure (see methods). The category indicates whether the interactions were previously reported in the literature or identified in the co-expression Ingenuity database (predicted) or not (new). The orientation indicates if the direction of the causal influence for the predicted interactions was the same as that in the structural network (right) or the opposite (reverse).

### Validation of the new inferred links

In order to assess the accuracy of our network analysis method and the validity of our T-cell activation network, we validated the newly inferred links as well some of the predicted ones in a new dataset of 16 healthy individuals. In addition, the validation of new links would probe that differences in the topology of the gene network have biological implications, at least at the gene expression level. In vitro assays were performed by stimulating PBMCs with the recombinant proteins involved in such links (the parent node), to assess their influence on the target gene (the child node) in terms of expression. We were able to experimentally validate the interaction between 7 of the 8 links assessed (87% validation), defining a statistical association between the levels of the parent and the child node (Table 3). We found a significant increase in CD28, IL12A and ITGA4 gene expression after 12 or 24 hours in cells stimulated with IL-10 (p<0.05, Table 3), validating the predicted arcs IL10–CD28 and IL10–ITGA4, and the new arc IL10–IL12A. Furthermore, JAG1 gene expression augmented after 24 hours in IL-4 stimulated cells (p = 0.017), validating the predicted edge JAG1–IL4. Finally, in CTLA-4 IgG stimulated cells JAG1, MX1 and PTPRC gene expression all increased significantly (p<0.05), validating the newly identified arcs CTLA4–JAG1, and CTLA4–MX1 and the predicted edge PTPRC–CTLA4. Thus, these results confirm the accuracy of the reconstructed T-cell activation network.

**Table 3:** Validation of the gene interactions in the T-cell activation network.

Stimulus	Target Gene	Gene Expression levels (mean (SD) ct value)			p value
		0 hours	12 hours	24 hours	
IL10	CD28	1.49E+01	1.14E+02	1.65E+02	<0.001^a^
		(1.25E+01)	(1.18E+02)	(1.43E+02)	<0.001^b^
	IL12A	3.15E+00	2.32E+01	3.54E+00	0.017^a^
		3.99E+00	(3.30E+01)	(3.27E+00)	
	ITGA4	6.72E+02	2.69E+03	2.60E+03	0.006^a^
		(8.22E+02)	(5.45E+03)	(2.59E+03)	<0.001^b^
IL4	HLA-DRA	3.20E+02	ND	9.46E+02	ns
		(2.92E+02)		(2.06E+03)	
	JAG1	1.64E-01	ND	5.55E-01	0.017^b^
		(9.38E-02)		(5.90E-01)	
CTLA4	JAG1	1.64E-01	ND	1.11E+00	<0.001^b^
		(9.38E-02)		(2.15E+00)	
	MX1	2.27E+01	ND	1.03E+02	0.045^b^
		(1.98E+01)		(1.76E+02)	
	PTPRC	2.67E-01	ND	7.50E-01	0.020^b^
		(1.58E-01)		(1.37E+00)	

Gene expression levels of target genes were assessed by real time PCR in PBMCs from 16 new healthy controls after stimulation with IL10, IL4 or CTLA4 (stimulus) for 12 to 24h. Results are described as the mean (SD) of the normalize ct value from real time PCR assays. Differences were assessed with the Mann-Whitney U test.

ND: not done; ns: not significant; ^a^p value comparing results at 0 to 12 hours; ^b^p value comparing results at 0 to 24 hours

### The topology of the network reveals pleiotropy of genes in biological functions

**Figure 2:**
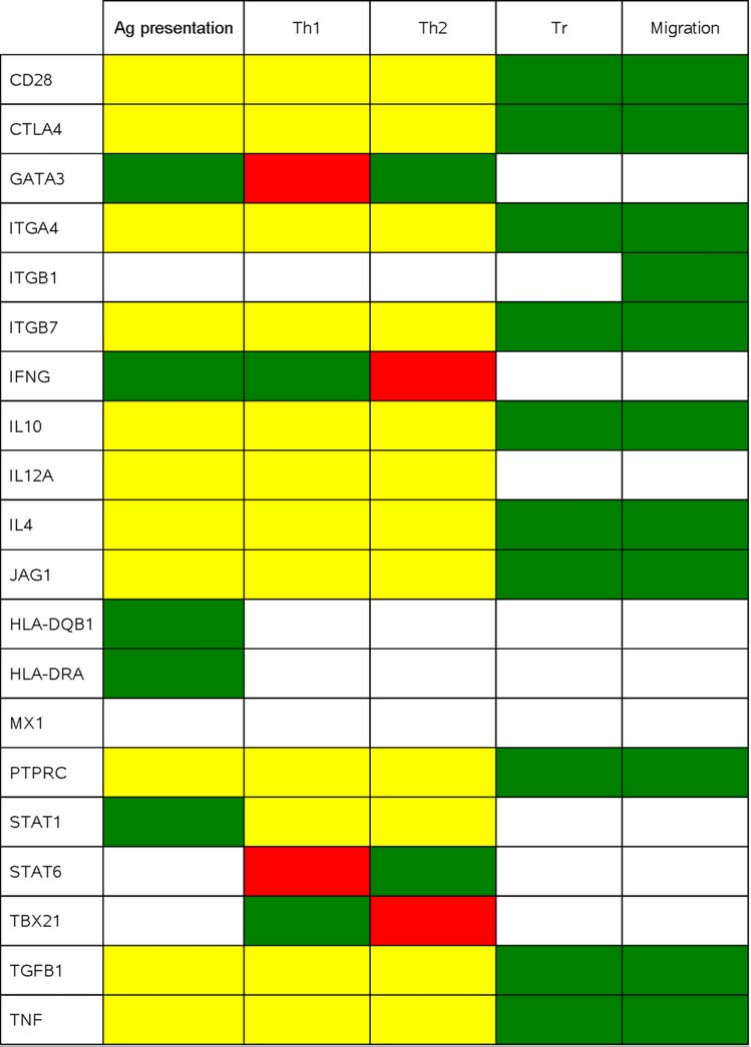
Network Analysis: Dependence matrix. The role of each gene in every T-cell activation function (antigen (Ag) presentation; Th1 differentiation; Th2 differentiation; Treg function; migration) based on the topology of the network is displayed using the following colour code: yellow: dual role (activator or inhibitor); green: full activator; red: full inhibitor; white: no influence.

We performed a qualitative analysis of the functional properties of the topology of the network [20], [21] using network analysis [22]. First we obtained the dependence matrix that assesses the role of every gene in a given function based on the definition of such function and the constraints imposed by the topology of our network (Figure 2). We found that the topology of the reconstructed network identified some of the biological function of the participating genes, such as the opposing roles of Th1-Th2 activity, as described by the stimulatory effect of IFNG and TBX21 in Th1 function, and of GATA3 and STAT6 in Th2 function, as well as by the inhibitory effect of IFNG and TBX21 in Th2 function, and of GATA3 and STAT6 in Th1 function (Figure 2). Indeed, the topological analysis identified HLA molecules as activators in the antigen presentation process and the role of CTLA4, IL-10, TGFß and JAG1 in Treg function. Moreover, the dependence matrix also highlighted the pleiotropic activity of many genes in T-cell activation, as represented by the dual role (in yellow) of several genes in the majority of the functions analyzed. In fact, the majority of the genes were activators of migration, indicating that the expression of adhesion molecules by T cells must be regulated after activation so that they may migrate and act in the target tissue. Furthermore, MX1 was included to assess the effect of IFN-ß therapy, although it does not influence T-cell activation, and we found that it did not affect the dependency matrix of the T-cell activation network, reinforcing the specificity of the analysis. However, the small size of the network and limitation of the network analysis process might prevent the right identification of the implication of every single gene in the functions studied, exemplified by the fact that in some cases the Cell Net Analyzer (CNA, see methods) was not able to discriminate a stimulatory or inhibitory effect and release a dual influence (yellow) or no influence (white) for a given gene. We identified the minimal cut sets (MCS, see methods) for each function in order to assess the structural robustness and fragility of the given function (Table 4). We found that the process of T-cell activation was very robust (robustness = 1, Table 4), mainly due to the selection of the genes critical for this process. Migration displayed an intermediate robustness (robustness = 0.58, Table 4) because most of the genes contribute to generate a specific pattern of adhesion molecule expression after T-cell activation. In contrast, Th1-Th2 and Tr function were not very robust (robustness = 0.27 and 0.21 respectively, Table 4), indicating that T-cell fate after activation is less fixed and that it is more sensitive to stochastic events or environmental signals [2].

**Table 4:** Network Analysis: Minimal cut-set analysis (MCS).

Cellular process	Fragility	Robustness	MCS
Ag presentation and co-stimulation	0	1	none
Th1 and Th2 differentiation	0.73	0.27	(JAG1, TNF) (CD28,JAG1)
			(IL4 TBX21 TNF)
			(ITGA4, IFNG, JAG1,STAT1)
			(IFNG, JAG1, STAT1, TGFB1)
			(ITGA4, IFNG, IL4, STAT1, TBX21)
			(IFNG, IL4, STAT1, TBX21, TGFB1)
			(GATA3, IFNG, IL12A, JAG1, STAT1)
			(GATA3, IFNG, IL12A, IL4, STAT1, TBX21)
Treg function	0.79	0.21	(CTLA4, JAG1, TNF)
			(CTLA4, CD28, JAG1)
			(CTLA4, IL4, TBX21, TNF)
			(CTLA4, ITGA4, IFNG, JAG1, STAT1)
			(CTLA4, IFNG, JAG1, STAT1, TGFB1)
			(CTLA4, ITGA4, IFNG, IL4, STAT1, TBX21)
			(CTLA4, IFNG, IL4, STAT1, TBX21, TGFB1)
Migration to tissues	0.42	0.58	(ITGA4)
			(IL10, TNF)
			(JAG1, TNF)
			(CD28, JAG1)
			(IL4, TNF)

### Differences in the T-cell activation network between healthy individuals and patients with Multiple Sclerosis

**Figure 3:**
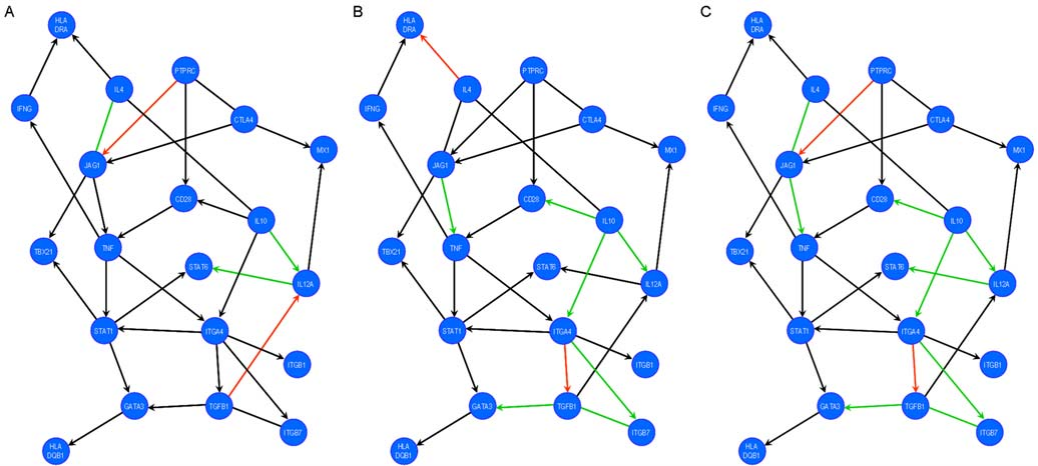
Comparison of the T-cell activation network between patients and controls. A) HC versus untreated MS patients; B) untreated MS patients versus MS patients treated with IFN-ß; C) HC versus patients treated with IFN-ß. Comparisons between gene interaction weights are described using the following colour code: black: no change; green: decreased; red: increased. See Table 4 for statistical analysis.

To quantify the importance of each interaction of the network in healthy controls, MS patients and MS patients treated with INF β, we calculated the Kullback-Leibler divergence (KL-divergence) of each interaction of the network for each diagnosis. To do that, we used the inferred topology of the network of T-cell activation and the gene expression levels of each of the groups (see methods). Thus, we obtained quantitative values that describe the behaviour of each interaction of the T-cell activation network under the different diagnosis. These values are indicative of the importance of the interaction, or to put it another way, they are indicative of the activation of that interaction. We found that the weight of some gene interactions differed in the two networks (Figure 3A represented by the colour of the arrow; Table 5). Accordingly, we found a significant decrease in the weight of the interaction between JAG1–IL4, IL10–IL12A, and IL12A–STAT6 in patients with MS (p<0.05), and a significant increase in the weight of the interaction between TGFB1–IL12A and PTPRC–JAG1 when compared to HC (p<0.05).

**Table 5:** Comparison in the weight of the interaction in the T-cell activation network between controls, MS patients and MS patients treated with IFN-ß.

Gene interaction		KL value			
Parent	Child	HC	MS	MS IFN-ß	p
TGFB1	IL12A	0.28 (0.11–0.48)	0.62 (0.35–0.90)	0.45 (0.03–0.60)	0.003^a^
ITGA4	TGFB1	0.30 (0.03–0.49)	0.19 (0.04–0.55)	0.99 (0.58–1.00)	<0.001^b^
					<0.001^c^
JAG1	IL4	0.58 (0.41–1.00)	0.28 (0.10–0.52)	0.27 (0.02–0.56)	0.012^a^
					0.008^c^
ITGA4	ITGB1	0.21 (0.07–0.48)	0.31 (0.13–0.35)	0.55 (0.07–0.99)	ns
IL4	HLA-DRA	0.34 (0.03–0.63)	0.22 (0.11–0.30)	0.47 (0.18–0.68)	0.001^b^
PTPRC	CD28	0.32 (0.16–0.47)	0.33 (0.16–0.69)	0.12 (0.03–0.85)	ns
TNF	IFNG	0.26 (0.16–0.46)	0.36 (0.12–0.62)	0.23 (0.06–0.61)	ns
JAG1	TNF	0.36 (0.24–0.60)	0.52 (0.19–0.68)	0.10 (0.00–0.37)	0.006^b^ 0.012^c^
CTLA4	JAG1	0.25 (0.12–0.52)	0.53 (0.23–0.61)	0.40 (0.08–0.59)	ns
STAT1	STAT6	0.22 (0.00–0.61)	0.32 (0.12–0.94)	0.43 (0.02–0.67)	ns
TGFB1	GATA3	0.39 (0.09–0.47)	0.18 (0.04–0.29)	0.00 (0.00–0.04)	<0.001^b^
					<0.001^c^
IL10	IL12A	0.36 (0.19–0.59)	0.07 (0.00–0.15)	0.00 (0.00–0.03)	<0.001^a^
					0.031^b^
					<0.001^c^
IL12A	STAT6	0.39 (0.12–0.79)	0.06 (0.00–0.23)	0.12 (0.00–0.25)	0.001^a^
					0.008^c^
ITGA4	STAT1	0.25 (0.05–0.64)	0.20 (0.01–0.37)	0.54 (0.00–0.62)	ns
IL4	IL10	0.22 (0.07–0.51)	0.10 (0.00–0.28)	0.05 (0.00–0.14)	ns
STAT1	GATA3	0.31 (0.09–0.53)	0.29 (0.12–0.49)	0.35 (0.00–0.41)	ns
IL12A	MX1	0.22 (0.03–0.69)	0.22 (0.04–0.32)	0.10 (0.00–0.36)	ns
IL10	CD28	0.23 (0.06–0.41)	0.12 (0.10–0.53)	0.03 (0.00–0.07)	0.043^b^
					<0.001^c^
TNF	ITGA4	0.23 (0.14–0.31)	0.36 (0.01–0.67)	0.14 (0.07–0.53)	ns
GATA3	HLA-DQB1	0.24 (0.06–0.46)	0.05 (0.01–0.33)	0.16 (0.00–0.37)	ns
STAT1	TBX21	0.30 (0.08–0.55)	0.23 (0.17–0.53)	0.20 (0.00–0.66)	ns
PTPRC	JAG1	0.13 (0.01–0.22)	0.24 (0.15–0.33)	0.39 (0.14–0.69)	0.048^a^
					0.011^c^
TGFB1	ITGB7	0.27 (0.02–0.36)	0.44 (0.21–0.60)	0.00 (0.00–0.00)	<0.001^b^
					<0.001^c^
IFNG	HLA-DRA	0.23 (0.00–0.53)	0.21 (0.03–0.29)	0.20 (0.15–0.39)	ns
PTPRC	CTLA4	0.19 (0.05–0.36)	0.04 (0.00–0.31)	0.06 (0.01–0.46)	ns
ITGA4	ITGB7	0.15 (0.00–0.38)	0.30 (0.03–0.41)	0.00 (0.00–0.00)	<0.001^b^
					0.007^c^
TNF	STAT1	0.37 (0.07–0.59)	0.19 (0.00–0.29)	0.35 (0.00–0.41)	ns
CTLA4	MX1	0.28 (0.01–0.43)	0.11 (0.01–0.40)	0.07 (0.00–0.22)	ns
CD28	TNF	0.12 (0.02–0.35)	0.14 (0.03–0.24)	0.21 (0.00–0.44)	ns
IL10	ITGA4	0.32 (0.16–0.39)	0.34 (0.20–0.72)	0.00 (0.00–0.09)	<0.001^b^
					<0.001^c^
JAG1	TBX21	0.22 (0.11–0.43)	0.06 (0.01–0.24)	0.15 (0.01–0.44)	ns

ns: not significant

a p value between HC and MS patients

b p value between MS patients and MS patients treated with IFN-ß

c p value between HC and MS patients treated with IFN-ß

Using the Kullback-Leibler (KL) divergence of links, we compared the differences between the control (HC), untreated MS patients (MS) and MS patients treated with IFN-ß (MS IFN-ß) networks. The results are described by the median (range) of the KL value. p values were adjusted using the Bonferroni method.

### Effect of immunotherapy in the T cell activation network

Because immunomodulatory treatments might exert their activity in a pleiotropic manner, we were interested in evaluating the effect of one of the therapies for MS in the network, such as INF-ß therapy. Our aim was to identify the functions targeted by IFN-ß in order to validate our approach to study immunotherapies at the systems level. Following the same procedure, we reconstructed the T-cell activation network using the gene expression levels from IFN-ß treated and untreated patients, and we compared the differences in interaction weight between pairs of genes (Figure 3B). When the IFN-ß treated and untreated patients were compared, we found a significant increase in the weight of the interaction between ITGA4–TGFB1 and IL4–HLA-DRA (p<0.001) and a significant decrease in the weight of the interaction between JAG1–TNF, TGFB1–GATA3, IL10–IL12A, IL10–CD28, TGFB1–ITGB7, ITGA4–ITGB7 and IL10–ITGA4 (p<0.005, Table 5). Thus, our results indicate that IFN-ß modifies different regions of the T-cell activation network, affecting different functions and thereby confirming its pleiotropic activity. While we identified the immunomodulatory effect of IFN-ß therapy in the T-cell activation network, we found that it did not restore their interaction weight to the baseline levels represented by the HC network (Figure 3C). Hence, IFN-ß therapy fails to completely restore the T-cell activation network indicating that this therapy is unable to completely normalize T cell function. This is in agreement with the clinical experience that IFN-ß therapy is only partially effective in MS [23].

We were also interested in identifying new therapeutic targets not addressed by IFN-ß therapy. Thus, we analyzed the differences between the HC and the IFN-ß treated patient networks. Ideally, a therapy will restore the functional state close to that of the healthy gene network and any deviation from such state can be considered as a therapeutic target [12]. The comparison between IFN-ß treated patients and HC networks (Figure 3C, Table 5) showed a significant increase in the weight of the gene interaction between the ITGA4–TGFB1, and the PTPRC–JAG1 genes (p<0.05) and a decrease in the weight of the interactions between JAG1–IL4, JAG1–TNF, TGFB1–GATA3, IL10–IL12A, IL12A–STAT6, IL10–CD28, TGFB1–ITGB7, ITGA4–ITGB7 and IL10–ITGA4 (p<0.05). Overall, we found that IFN-ß therapy failed to restore the balance between pro-inflammatory cytokines and Treg function. Pro-inflammatory cytokines, such as TNF or IL12A, the T-cell activation molecule CD28, the regulatory molecules IL10, TGFB1 or the adhesion molecules ITGA4 and ITGB1 have all been evaluated previously as therapeutic targets for autoimmune diseases, including MS [24]–[29]. Hence, we focused on the JAG1 gene as a potential target for new therapies to treat autoimmune diseases. JAG1 plays a critical role in our network since it was consistently modified in the disease state and its interactions were almost unmodified by IFN-ß therapy, making it an excellent therapeutic target in our model, even if JAG1 mRNA levels were not significantly different between patients and controls (Table S1). Moreover, JAG1 has recently being identified as a candidate gene for MS [30].

### Validation of JAG1 as a therapeutic target in Multiple Sclerosis

**Figure 4:**
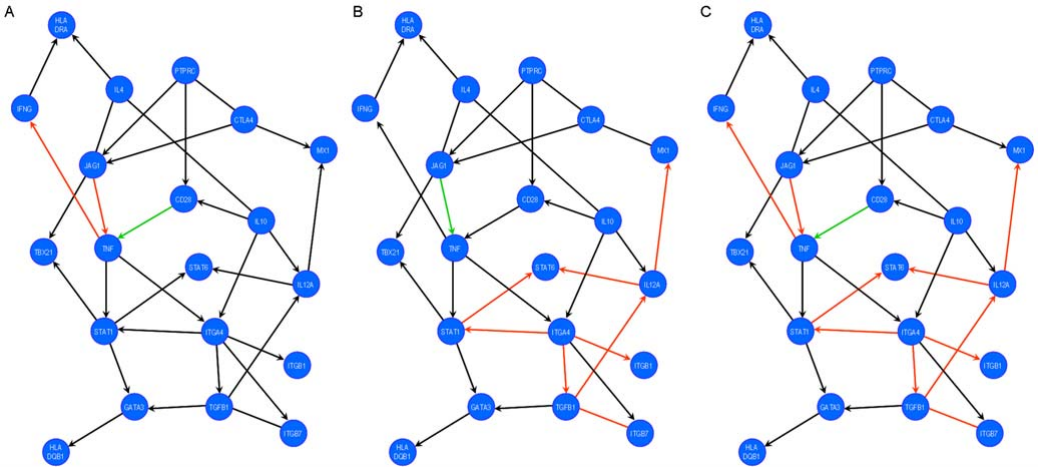
Comparison of the T-cell activation network after in vitro stimulation with jagged1, IFN-ß or Jagged1+IFN-ß: A) untreated patients versus stimulation with the Jagged1 agonistic peptide for 24 h; B) untreated patients versus stimulation with IFN-B for 24 h; C) untreated patients versus stimulation with JGA1+IFN- ß for 24 h. Comparisons between gene interaction weights are described using the following colour code: black: no change; green: decreased; red: increased. See Table 5 for statistical analysis.

Notch signalling in the adult immune system has several functions depending on the ligand involved [31], [32]. Jagged1 signalling promotes Treg function whereas Delta1 promotes pro-inflammatory responses. In order to validate the JAG1 gene and its protein jagged1 as a therapeutic target, first we assessed the effect of stimulating human PBMCs from MS patients *in vitro* with a Jagged1 peptide agonist [33]. We used the HES5 and MX1 genes as reporters of the Jagged1-Notch pathway and of the IFN-ß-IFNR1 pathway, respectively. Accordingly, PBMCs cultures stimulated with either the Jagged1 peptide agonist or IFN-ß displayed a significant increase in HES5 or MX1 gene expression (Supplementary Table S4). Moreover, the Jagged1 stimulated network differed from the untreated and the IFN-ß treated network (Table 6, Fig 4A). Compared to the network from untreated patients, Jagged1 therapy induced an increase in the interaction weight between JAG1-TNF and the downstream interaction TNF-IFNG, as well as a decrease in the interaction weight between CD28-TNF (Figure 4A). Thus, our network analysis was able to capture the biological effect of Jagged1-Notch signalling in T-cell activation. Our results suggested that the Jagged1-Notch pathway modulates Th1 function. Exposure of PBMCs from untreated patients to IFN-ß *in vitro* yielded a network similar to that obtained *ex-vivo* from IFN-ß treated patients (Figure 3B and 4B), although some quantitative differences in the interaction weight between the pairs of genes involved in the effect of IFN-ß were detected. Indeed, IFN-ß therapy impaired the effect of JAG1 on TNF that is required for Jagged1 to suppress Th1 function in our model. Finally, the combination of Jagged1 and IFN-ß treatment indicates a synergistic effect of both therapies (Table 6, Fig 4C), and the effect of each therapy could be identified in the different network interactions, which might produce different functional effects. Moreover, Jagged1 therapy was able to recover the effect of JAG1 on TNF that was lost with IFN-ß therapy, suggesting that this combined therapy will maintain the Th1 function suppression induced by Jagged1.

**Table 6:** Comparison in the weight of the interaction in the T-cell activation network between jagged1, IFN-ß and jagged1 plus IFN-ß treatments in vitro.

Gene interaction		KL value				
Parent	Child	Basal	Jagged1	IFN-ß	Jagged1+IFN-ß	p value
TGFB1	IL12A	0.02 (0.00–0.04)	0.02 (0.01–0.04)	0.09 (0.02–0.15)	0.08 (0.06–0.11)	<0.001^b^
						<0.001^c^
ITGA4	TGFB1	0.03 (0.01–0.05)	0.03 (0.01–0.04)	0.09 (0.02–0.15)	0.08 (0.02–0.13)	<0.001^b^
						<0.001^c^
JAG1	IL4	0.04 (0.01–0.07)	0.02 (0.00–0.04)	0.03 (0.00–0.05)	0.03 (0.00–0.05)	ns
ITGA4	ITGB1	0.01 (0.00–0.01)	0.00 (0.00–0.01)	0.12 (0.04–0.21)	0.14 (0.09–0.18)	<0.001^b^
						<0.001^c^
IL4	HLA-DRA	0.11 (0.06–0.16)	0.10 (0.07–0.13)	0.13 (0.05–0.22)	0.13 (0.02–0.24)	ns
PTPRC	CD28	0.00 (0.00–0.00)	0.00 (0.00–0.00)	0.00 (0.00–0.00)	0.00 (0.00–0.00)	ns
TNF	IFNG	0.00 (0.00–0.00)	0.02 (0.00–0.05)	0.00 (0.00–0.00)	0.05 (0.00–0.11)	<0.001^a^
						<0.001^c^
JAG1	TNF	0.03 (0.01–0.05)	0.09 (0.01–0.18)	0.02 (0.01–0.03)	0.20 (0.02–0.41)	0.014^a^
						0.047^b^
						0.004^c^
CTLA4	JAG1	0.00 (0.00–0.00)	0.00 (0.00–0.00)	0.00 (0.00–0.00)	0.00 (0.00–0.00)	ns
STAT1	STAT6	0.00 (0.00–0.00)	0.00 (0.00–0.00)	0.01 (0.00–0.02)	0.01 (0.00–0.02)	0.031^b^
						0.045^c^
TGFB1	GATA3	0.15 (0.08–0.22)	0.12 (0.08–0.16)	0.13 (0.10–0.17)	0.16 (0.06–0.26)	ns
IL10	IL12A	0.00 (0.00–0.00)	0.00 (0.00–0.00)	0.00 (0.00–0.00)	0.00 (0.00–0.00)	ns
IL12A	STAT6	0.01 (0.00–0.02)	0.01 (0.00–0.03)	0.04 (0.01–0.07)	0.13 (0.12–0.14)	<0.001^b^
						<0.001^c^
ITGA4	STAT1	0.02 (0.01–0.04)	0.02 (0.01–0.03)	0.05 (0.01–0.09)	0.06 (0.04–0.08)	<0.001^b^
						<0.001^c^
IL4	IL10	0.00 (0.00–0.00)	0.00 (0.00–0.00)	0.00 (0.00–0.00)	0.00 (0.00–0.00)	ns
STAT1	GATA3	0.06 (0.01–0.11)	0.02 (0.07–0.12)	0.02 (0.01–0.04)	0.02 (0.07–0.1)	ns
IL12A	MX1	0.01 (0.00–0.01)	0.00 (0.00–0.01)	0.01 (0.00–0.03)	0.03 (0.02–0.04)	<0.001^b^
						<0.001^c^
IL10	CD28	0.00 (0.00–0.00)	0.00 (0.00–0.00)	0.00 (0.00–0.00)	0.00 (0.00–0.00)	ns
TNF	ITGA4	0.00 (0.00–0.00)	0.00 (0.00–0.01)	0.01 (0.00–0.01)	0.00 (0.00–0.01)	ns
GATA3	HLA-DQB1	0.07 (0.03–0.1)	0.05 (0.01–0.11)	0.08 (0.02–0.14)	0.13 (0.08–0.18)	ns
STAT1	TBX21	0.24 (0.15–0.33)	0.20 (0.14–0.26)	0.20 (0.15–0.24)	0.22 (0.09–0.35)	ns
PTPRC	JAG1	0.00 (0.00–0.00)	0.00 (0.00–0.00)	0.00 (0.00–0.00)	0.00 (0.00–0.00)	ns
TGFB1	ITGB7	0.06 (0.02–0.09)	0.07 (0.03–0.10)	0.12 (0.07–0.18)	0.17 (0.07–0.27)	<0.001^b^
						<0.001^c^
IFNG	HLA-DRA	0.12 (0.09–0.15)	0.16 (0.09–0.24)	0.18 (0.08–0.27)	0.10 (0.03–0.16)	ns
PTPRC	CTLA4	0.00 (0.00–0.00)	0.00 (0.00–0.00)	0.00 (0.00–0.00)	0.00 (0.00–0.00)	ns
ITGA4	ITGB7	0.00 (0.00–0.00)	0.00 (0.00–0.00)	0.00 (0.00–0.00)	0.00 (0.00–0.00)	ns
TNF	STAT1	0.01 (0.01–0.02)	0.01 (0.00–0.01)	0.01 (0.00–0.01)	0.03 (0.00–0.05)	ns
CTLA4	MX1	0.00 (0.00–0.00)	0.00 (0.00–0.00)	0.00 (0.00–0.00)	0.00 (0.00–0.00)	ns
CD28	TNF	0.02 (0.01–0.03)	0.00 (0.00–0.01)	0.02 (0.00–0.03)	0.00 (0.00–0.01)	<0.001^a^
						<0.001^c^
IL10	ITGA4	0.00 (0.00–0.00)	0.00 (0.00–0.00)	0.00 (0.00–0.00)	0.00 (0.00–0.00)	ns
JAG1	TBX21	0.06 (0.03–0.09)	0.06 (0.02–0.11)	0.09 (0.04–0.14)	0.04 (0.02–0.07)	ns

ns: not significant

a p value between basal and Jagged 1

b p value between basal and IFN-ß

c p value between basal and Jagged1+IFN-ß

Using the Kullback-Leibler (KL) divergence of links, we compared the differences from *in vitro* assays in the interaction weight between pairs of genes using PBMCs from untreated patients that were stimulated with either the jagged1 agonist peptide, IFN-ß or Jagged1 plus IFN-ß. Results are described as the median (range) of the K-L value. p values were adjusted using the Bonferroni correction for multiple testing.

**Figure 5:**
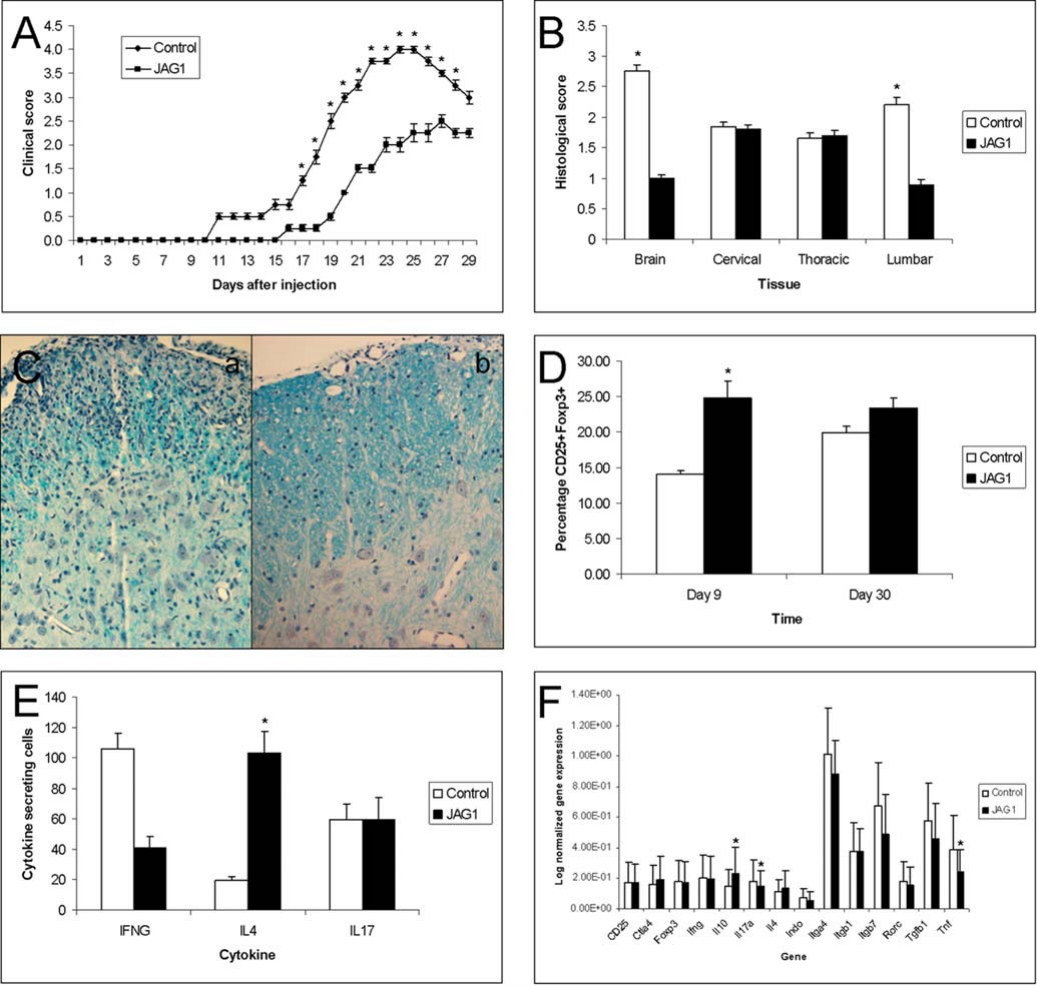
Validation of Jagged1 as a therapeutic target in the animal model of MS: C57B6 mice (n = 60) were immunized with MOG_35–55_ and treated with Jagged1 agonist peptide i.p. (n = 30) or placebo (n = 30) from day 0 to day 30 in two different experiments. Twelve animals (6 from each treatment group) were sacrificed by day 9 in order to perform immunological studies. Animals treated with the Jagged1 agonist peptide have a clinical (A) and histological (B) score lower than placebo animals. C) Representative spinal cord sections from placebo (a) and jagged1 (b) treated animals stained with Luxol-fast blue showing a decrease in the presence of inflammatory infiltrates and the extend of demyelination in the Jagged1 treated animals. FACS analysis assessing the percentage of CD25+Foxp3+ Treg cells (D) ELISPOT studies assessing the in vitro secretion of Th1 (INFγ), Th2 (IL-4) and Th17 (IL-17) cytokines (E) and real-time PCR (F) studies in splenocytes from Jagged1 treated and untreated mice. D) Mice treated with Jagged1 peptide have higher percentage of Treg cells than placebo animals by day 9 p.i. (p = 0.032), and such difference disappeared by day 30. E) Splenocytes from Jagged1 treated mice expressed higher levels of IL-4, lower levels of IFNγ and similar levels of Il-17 than placebo animals at day 9 p.i. F) Jagged1 treated animals expressed higher levels of IL-10 and lower levels of TNFα and IL-17 than placebo animals at day 9 p.i. Results are expressed as the mean±SEM

In order to confirm the results from our network analysis, we evaluated the effect of the Jagged1 agonist peptide in the animal model of MS. C57B6 mice immunized with MOG_35–55_ peptide were treated with the Jagged1 peptide i.p. from day 0 to day 30 and this produced a milder progression of the disease (Figure 5A) and lower histological scores (Figure 5B and 5C) than in placebo animals. Hence, Jagged1-Notch signalling appears to exert an immunomodulatory effect in brain autoimmunity. We studied the possible mode of action of Jagged1 therapy by assessing several immune responses involved in autoimmunity. In addition, we tested the *in vivo* effect of Jagged1 therapy in the genes of the T-cell activation network. Previous studies suggested a role for Jagged1-Notch signalling in promoting Treg and Th2 function [31]. We found that by day 9 after immunization, animals treated with Jagged1 peptide have increased percentage of CD25+Foxp3+ cells that placebo animals (21.6% compared to 13.6%, p = 0.032; Fig. 5D) and such differences were lost by day 30 (Fig. 5D). In addition, we also found that by day 9 after immunization, Jagged1 treated animals have significant higher numbers of Th2 cells (IL-4 secreting cells; p = 0.032) and a trend for decreased numbers of Th1 cells (IFNγ secreting cells; p = 0.056) than placebo animals, and not changes in numbers of IL-17 secreting cells (Fig. 5E). By day 30, differences were lost and frequency of such populations were similar between Jagged1 and placebo treated animals (data not shown). We also analyzed the effect of Jagged1 peptide therapy in gene expression levels in splenocytes by day 9 and 30 after immunization. We found that by day 9 p.i. Jagged1 treated animals have increased gene expression levels of IL-10 (p = 0.029) and decreased levels of TNFα and IL-17a (p<0.05 in both cases, Fig. 5F and Table S5). By day 30, we found a decrease in TGFß1, TNFα, and ITGB7 gene expression levels in animals treated with Jagged1 when compared with untreated animals (p<0.05 in all cases; Table S5). These results might suggest that Jagged1-Notch signalling promotes Treg and Th2 responses and suppress Th1 function. Thus, our findings indicate that the Jagged1-Notch pathway may be a therapeutic target to treat MS and other autoimmune diseases. Moreover, our network approach allowed us to predict a mechanism of action that was not expected from previous biological knowledge.

## Discussion

In the present study we have provided proof of concept that a gene network analysis approach is feasible to study human systems and diseases, providing valuable information about the complex interactions involved in biological process and in complex diseases. This is important since most systems biology studies have been applied to lower organisms and its application to higher animals and humans has been restricted by the lack of biological knowledge, technological and analytical tools, as well as by the higher degree of complexity of such organisms. Biological functions, as well as complex traits and diseases, can only rarely be attributed to an individual molecule. On the contrary, complex interactions between dozens of molecules lead to a specific biological function [34], and altering the relationships between these elements may disrupt the activity in such systems. Network analysis has emerged as a powerful tool to understand complex intercellular interactions that contribute to the structure and function of living systems [1] and it can be used to study complex traits and diseases in order to discover new therapies [35], [36]. Indeed, the application of a Bayesian approach to define cell networks has been successfully used and as well as to infer classic, well understood signalling networks [37]. Such an approach has also provided new insights into specific systems that have not been previously identified through hypothesis-directed research [17].

In our network analysis, we identified 31 interactions between 20 genes acting in the immune system of which, 18 were predicted either from the literature or by bioinformatics analysis of co-expression databases. However, the other 13 interactions were new and had not been predicted using bioinformatics strategies. These latter interactions could be more specific for autoimmunity such that our experimental dataset from patients suffering an autoimmune disease was informative to highlight the importance of these interactions when compared to the healthy state. It must to be remembered that the gene interactions identified in our network does not mean that they are direct molecular interactions and intermediary molecules might account for such effect. Indeed, among the many interactions between the genes in our network, we were able to identify either the most robust or the ones that in the specific condition we are studying (MS) are most relevant, implying that some others could be missed for our analysis, even if they have been biologically demonstrated. The validation of the majority of these interactions, both the predicted and new interactions, indicates that our approach was sufficiently powerful and accurate to identify true gene interactions in human cells. In addition, the validation of new gene interactions indicates that differences in gene interaction weight have biological implications, at least at the gene expression level. The high validation rate was due to the balanced design between the number of genes studied, the use of accurate quantitative methods and the biological knowledge available in the literature, as well as benefiting from the use of system biology methods such as Bayesian inference, previously validated in other settings. In addition, in the majority of the cases we were also able to correctly identify the direction of the interactions. Identifying the direction of an interaction is an important step in reverse network engineering and requires larger datasets for network inference [17]. Although we use a mixed cell population such as PBMCs, our results indicates that the network analysis was able to identify the contribution of every gene to the T cell activation process, which is a multicellular process by definition implying antigen presenting cells and T cells. Finally, the qualitative analysis of the network using CNA shows that our method was able to identify a network topology, which is related with the biological functions involved. This analysis was more an attempt to confirm the influence of network topology in biological functions even if such analysis was limited by the size of our network and was not able to predict all functions studied (e.g. the role of IL-4 in Th2). Thus, our results suggest that the reconstruction of biological networks from experimental data is a feasible strategy to study human system and diseases.

Biological networks are robust [1], and we predicted that the differences between the healthy and disease state for complex and polygenic disease would not be based on the deletion of nodes (genes or proteins) or even links (gene or protein interactions), but rather in more subtle changes in the weight of the interactions. For this reason we were more interested in performing a quantitative analysis of the network than in the topological differences between such networks. Our quantitative analysis of the network that controls T-cell activation revealed several significant differences between the healthy state and MS. MS is an inflammatory disease in which T-cell activation is dysfunctional [6] and regulatory T cell function is impaired [38]. The comparison between the T-cell activation networks from HC and MS patients showed an overall decrease in the activity (defined here by the change in the weight of the interaction between pair of genes) of Treg and Th2 function, and an increase in Th1 activity. For example, JAG1 constitutes an instructive signal for Th2 differentiation by inducing GATA3 and by directly regulating IL4 gene transcription [39]. Thus, the reduction in the weight of the JAG1–IL4 gene interaction in the MS patient network indicates a decrease in Th2 differentiation. Furthermore, the reduced weight of the IL12A–STAT6 interaction also suggests diminished Th2 differentiation due to the fact that STAT6 plays a central role in exerting IL4 mediated biological responses [40] and IL12 is important in the regulation of the Th2 response [41]. Indeed, a reduction in the weight of the IL10–IL12A interaction indicates a decrease in the suppression mediated by IL-10 over Th1 function [42]. On the other hand, an increase in the weight of the TGFB1–IL12A and PTPRC–JAG1 interactions suggests more pronounced Th1 activity in patients with MS. IL12 is required for the induction of IFNγ and PTPRC induces the expression of TNFα [43], which can stimulate cell proliferation via Jagged1 [44]. Thus, our results identify JAG1 and IL12A as critical genes in the pathogenesis of MS. IL12 plays a central role in brain autoimmunity [45] and recent genetic studies have associated JAG1 to MS susceptibility [30]. Of course, several other genes not included in our study may play a role in the pathogenesis of MS, but our results indicate that even using a small set of genes, a quantitative network analysis is able to identify functional differences between healthy and disease states.

Moreover, we show that a quantitative network analysis provides valuable information about how therapies work at a system level, showing that in most cases they exert a pleiotropic activity that is difficult to capture in single molecule assays. But more importantly, our approach allowed us to identify new therapeutic targets that are not modified by current therapies, based on the quantitative changes in the network produced by the disease. Current immunotherapies for autoimmune diseases such as interferon beta, glatiramer acetate or natalizumab exert their action through pleiotropic activities that are poorly understood [46]. In our study, the increase in the weight of the ITGA4–TGFB1 interaction and the decrease in weight of the JAG1–TNF, TGFB1–GATA3, IL10–CD28, TGFB1–ITGB7, ITGA4–ITGB7 and IL10–ITGA4 interactions in patients treated with IFN-beta when compared with untreated MS patients and HC, suggest a direct influence of IFN-ß on these pathways. The decrease in the weight of the JAG1–TNF, TGFB1–GATA3 and IL10–CD28 interactions support the idea that IFN-ß activity involves the suppression of T cell proliferation, the induction of Th2 cytokine production, inhibition of Th1 cytokine production and inhibition of monocyte activation [47]. Also, the changes in the links involving the adhesion molecules ITGA4, ITGB1 and ITGB7 confirm the role of IFN-ß treatment in decreasing the surface expression of adhesion molecules, which reduces the migratory potential of T cells [48]. The *in vitro* validation of the IL10–IL12A interaction deserves special mention, as it suggests that IFN-ß affects this pathway. However, based on our analysis, IFN-ß therapy failed to restore such an interaction to the normal levels in the healthy state. Indeed, differences in the weight of the JAG1–IL4, IL12A–STAT6 and PTPRC–JAG1 interactions in treated or untreated MS patients compared with HC would suggest that IFN-ß treatment has no effect on these pathways and as such, they may be considered as novel therapeutic targets. Taking into account that JAG1 seems to be involved in the genetic susceptibility of MS, its role in the immune system, and the failure of IFN-ß treatment to modulate its function, we propose JAG1 as a new therapeutic target in the treatment of the MS.

As a proof of concept that network analysis might identify valuable therapeutic targets, we validated the Jagged1-Notch pathway as a therapeutic target for MS. The Notch pathway exerts several activities in the developing, as well as in the mature peripheral immune system [31]. The outcome of Notch signalling depends on the ligand involved and accordingly, it has been shown that Jagged1 activates Treg and Th2 function whereas Delta1 promotes the Th1 response. We were able to demonstrate that treating immune cells from patients with MS with an agonistic peptide of Jagged1 *in vitro* modulates the T-cell activation network, mainly suppressing Th1 function (represented by the strong effect of JAG1 over TNF and the downstream effect of TNF on IFNG). Interfering with Jagged1 has been shown to suppress IFNγ production by dendritic cells [49]. Moreover, we found that IFN-ß and Jagged1 therapy exerted a synergy in vitro. In our network analysis we were able to identify the individual and non-overlapping effects of both therapies and the predominant role of Jagged1 in suppressing Th1 activation, overcoming the suppressor effect of IFN-ß therapy on the JAG1-TNF interaction. Finally, we were able to identify the therapeutic potential of the Jagged1-Notch pathway in the animal model of MS, confirming its immunomodulatory effect. We found that Jagged1-Notch signalling enhances Treg and Th2 function and suppressed Th1 function in mice suffering an autoimmune disease. Although we found no effect of Jagged1 therapy in Th-17 subset (no changes in levels of secreted IL-17 in the ELISpot assays), indirect evidence might suggest that Jagged1 might influence also this function, since critical molecules for Th-17 induction were modified by Jagged1 therapy at the RNA level such as IL17a, TNFα and TGFß. Finally, it has been described that Notch signalling in MS brains might contribute to failure of regenerative process [50]. We found no evidences that treating animals with an agonistic peptide of Jagged1 might impair recovery for EAE. However, further studies will be required to fully validate the therapeutic opportunities associated with the Jagged1-Notch pathway in autoimmune diseases, mainly considering the critical and pleiotropic functions that this pathway plays in many cell types that might lead to undesired side effects.

In summary, our results suggest that a quantitative network approach is a useful tool in medicine to understand complex diseases and discover new therapeutic targets. In particular, the Jagged1-Notch pathway seems to be a good candidate to participate in the susceptibility and therapy of MS. Hence, a more profound study of its regulation may help to understand its implication in MS and its potential for therapy.

## Methods

### Human subjects and biological assays

#### 1. Human subjects

We studied 52 patients with MS [51] and 52 sex and age matched healthy controls (HC) from the same population with no history of autoimmune diseases. For validation assays of the identified links in the network we used a second set of 16 healthy individuals. For the in vitro assays with PBMCs for testing the role of Jagged1 peptide and IFNB, we used a new set of 24 patients with MS that were not receiving immunomodulatory therapy. The demographic and clinical data from the subjects are shown in Supplementary Table S1. Patients were recruited by their neurologist after obtaining written informed consent. This study was approved by the Institutional Review Board at the University of Navarra.

#### 2. RNA extraction, probes and rtPCR

Peripheral blood mononuclear cells (PBMCs) were isolated using Ficoll-Paque (Pharmacia Biotech) and they were immediately submerged in RNAlater RNA Stabilization Reagent (Qiagen) to preserve the gene expression patterns. Total RNA was isolated using the RNeasy Mini Kit (Qiagen), removing DNA with the RNase-Free DNase Set (Qiagen), and the High-Capacity cDNA Archive Kit (Applied Biosystems) was used to synthesise cDNA from the total RNA. Primer sequences and target-specific fluorescence-labelled TaqMan probes were purchased from Applied Biosystems (TaqMan Gene Expression Assays, Supplementary Table S2).

Quantitative real-time PCR (rt-PCR) was performed with the DNA Engine Opticon2 (MJ Research). Each sample was run in triplicate and the target and endogenous control gene were amplified in different wells on each plate. We used GAPDH and B2M as controls because both genes were described as a good choice to normalize leukocyte expression levels [52]. Cycle threshold (c(t)) values were acquired with the Opticon Monitor 2.01 software (MJ Research) and using this information, we subtracted the baseline signal as the average of the fluorescence measured from cycle 1 to 40 and we set the c(t) line to a standard deviation of 1.00. The normalized gene expression was calculated using the Q-Gene software application [53], which does not assume that the PCR amplification efficiencies of target and reference genes are equivalent. Real-time PCR efficiencies (E) were calculated by amplifying a series of 2-fold dilutions of each factor. The mean c(t) values were plotted against the log of the amount of cDNA added. The linear graphs obtained (correlation coefficients>0.99) were used to calculate the corresponding E. The mean normalized expression was given by calculating the average c(t) values of the target and reference triplicates. Single gene expression values and the comparison between groups are shown in supplementary Table S1.

#### 3. Validation of the new inferred links

Freshly isolated PBMCs, from a new set of 16 HC not previously used in the network construction, were stimulated with 20 ng/ml recombinant human IL10 (R&D Systems), 4 ng/ml recombinant human IL4 (R&D Systems) or 40 ng/ml recombinant human CTLA4/Fc chimera (R&D Systems) for 12 and 24 hours, or left unstimulated as controls. At the end of the assay, RNA was isolated from the cells and gene expression was measured by rtPCR as described above.

#### 4. In vitro Jagged1 validation assays

Jagged1 validation assays were performed using PBMC from patients with MS untreated and stimulated *in vitro* with 20 µg/ml of human jagged1 peptide 188–204 (CDDYYYGFGCNKFCRPR; Sigma-Aldrich) [33], or with 1.000 u/ml of recombinant human INF-β (PBL Biomedical Laboratories) or both. Cells were incubated for 24 h and RNA was extracted and quantified by real time PCR as described before. KL-divergence values were calculated for each group in the T-cell activation network as described above.

#### 5. Experimental Autoimmune encephalomyelitis

Female C57B6 mice obtained from Charles River (6–8 weeks old; 20 gr. body weight) were immunized in the lateral flank with a 100 µl emulsion of saline and incomplete Freund́s adjuvant containing 100 µg Myelin Olygrodendrocyte Glycoprotein (MOG_35–55_) peptide from Sigma (Germany) supplemented with 4 mg/ml *Mycobacterium tuberculosis* (H37Ra strain from Difco, Detroit, MI). The animals were weighed on a daily basis and inspected for clinical signs of experimental autoimmune encephalomyelitis (EAE) by a blind observer as described previously [54]. Animals were treated with 1 mg/day of human jagged1 peptide 188–204 (CDDYYYGFGCNKFCRPR; Sigma-Aldrich) [33] beginning at the day of immunization until day 30 post-immunization. Disease severity was assessed according to the following scale: 0 = normal; 1 = limp tail; 2 = mild paraparesis of the hind limbs, unsteady gait; 3 = moderate paraparesis, voluntary movements still possible; 4 = paraplegia or tetraparesis; 5 = moribund state. Data shown for the EAE studies are representative of two independent experiments performed with the number of animals indicated. The University of Navarra Committee for Animal Care approved the entire animal studies carried out.

Histological evaluation was performed on paraformaldehyde-fixed, paraffin embedded sections of the brain and spinal cord, as described previously [54]. Sections (10 µm thick) were stained with haematoxylin and eosin (H&E) and with Luxol fast-blue to assess inflammation and demyelination. We examined 20 consecutive sagittal sections from each region examined (brain, cervical, thoracic and lumbar spinal cord) of every animal in the study. Semi-quantitative histological evaluation for inflammation and demyelination was carried out and scored blindly using the following scale: 0, normal; 1, 1-3/section perivascular cuffs with minimal demyelination; 2, 3-10 perivascular cuffs/section accompanied by moderate demyelination; 3, widespread perivascular cuffing, extensive demyelination with large confluent lesions.

#### 6. Flow Cytometry analysis

Cells were phenotyped by three-colour flow cytometry (FACSaria) according to the expression of CD25 and Foxp3 using the mouse regulatory T cell staining kit from Ebiosciences, UK. We analyzed the splenocytes from naïve or treated C57B6 mice suffering EAE at day 9 (n = 6 placebo and 6 Jagged1 treated mice) and day 30 (n = 24 placebo and 24 Jagged1 treated mice) postinjection (p.i.).

#### 7. ELISpot

We assessed the secretion of IFN-γ, IL-4 and IL-17 in splenocytes from naïve or treated C57B6 mice suffering EAE at day 9 using the mouse-IFNγ and mouse-IL4 ELISpot plus kit from Mabtech (Mabtech, US), and the mouse-IL17 ELISpot from Ebioscience (Ebioscience,UK) according to the manufacturer's instructions. Antigen stimulation was carried out with the immunizing antigen (MOG_35–55_ 100 µg/ml) for 48 h and we used either PHA or ConA (5 µg/ml) as a positive control for Th1 and Th2, or for Th17, respectively. ELISpot quantification was performed with the Immunospot S4 Pro Analyzer (Cellular Technology Ltd, US).

### Network Analysis

#### 1. Ingenuity Pathways Knowledge Base analysis

Because Bayesian algorithms might provide several different results with the same experimental dataset, feeding the algorithm with a template of the network will diminish the number of different outputs and increase their accuracy. Thus, in order to obtain a template (*structural network*) of the gene network that controls the T-cell activation, we used biological knowledge from co-expression data available at the Ingenuity database, using the Ingenuity Pathways Knowledge Base (Ingenuity Systems Inc. Redwood City, USA). We were interested in evaluating five critical processes in T-cell activation, namely: 1) antigen presentation and co-stimulation, 2) Th1 differentiation; 3) Th2 differentiation; 4) T regulatory (Treg) function; and 5) migration to tissues. Genes were distributed in five biological functions based in biological knowledge (Table S2) as follows: 1) antigen presentation, signalling threshold modulation and co-stimulation-HLA-DRA, HLA-DQB1, PTPRC (CD45) and CD28; 2) Th1 differentiation-TBX21 (T-bet), STAT1, IFNG, TNFA and IL12A; 3) Th2 differentiation-GATA3, STAT6, IL4, JAG1; 4) Treg function-CTLA4, IL10, TGFB1 and JAG1; and 5) Migration-ITGA4, ITGB1, ITGB7. There is significant biological knowledge regarding each of these genes and they have all been implicated in autoimmune diseases, including MS (see supplementary Table S2 and S3).

#### 2. Bayesian network algorithm

Gene expression data from MS patients and HC were made discrete in two intervals with the same number of associated cases. To model the T-cell activation network, we performed structural learning using a Tabu Search [55], based on the experimental data and retaining the structure of the network obtained in the Ingenuity database search described above. Because the inference of the Bayesian network does not accept network cycles, the *structural network* template was introduced after manually curated the presence of such cycles by changing the orientation of links in order to maintain the link and avoid cycles. During this procedure, no links were removed. The overall approach of the Tabu Search is to avoid entrainment in cycles by forbidding or penalizing moves that, in the next iteration, take the solution to points in the solution space previously visited [55]. The Tabu Search algorithm constructs a graphic in which the nodes represent the measured gene levels and the arcs represent statistically meaningful relationships and the dependency between these genes. A Tabu Search was performed using a tabu list size of 10 and a structural complexity influence of 1. Bayesian analysis was processed using the BayesiaLab 3.3 software (Bayesia SA. Laval Cedex, France). The algorithm considered links as arcs (links in which the orientation of the connection cannot be changed without changing the probabilistic relations encoded) if the available information allow the algorithm to define the direction of the interaction, or as edges (links in which the orientation can be inverted) if the algorithm was not able to definitively confirm a unique direction for the interaction.

#### 3. Qualitative analysis of the network

We took advantage of tools developed for metabolomics studies to assess the functional consequences of the network topology [20], [21]. The Cell Net Analysis (CNA) approach and CNA 6.0 software (Steffen Klamt, Max-Planck Institute, Magdeburg, Germany) was used to measure the overall network function since gene expression patterns could be considered stable states. We first evaluated the dependence matrix in order to assess the functional consistency of the reconstructed network. We then identified the minimal cut sets (MCS) [22] required for the loss of a defined cell function. The concept of minimal cut sets has been introduced to study structural fragility and to identify knock-out strategies in biochemical reaction networks [22]. In this study, a MCS was defined as a minimal set of interactions whose removal blocks cell function (e.g. Th1 differentiation). We defined the maintenance of a given function based on biological knowledge using logical rules as follow: 1) Antigen presentation and co-stimulation: CD28 AND PTPRC AND (HLA-DQB1 OR HLA-DRA) NOT (CTLA4 OR JAG1OR (CTLA4 AND JAG1) OR IL10 OR TGFB1 OR (IL10 AND TGFB1) OR (CTLA4 OR JAG1 OR (CTLA4 AND JAG1) AND (IL10 OR TGFB1 OR (IL10 AND TGFB1)))); 2) Th1 differentiation: INFG AND IL12A AND STAT1 AND TBX21 AND TNF NOT (IL4 AND STAT6 AND GATA3 AND JAG1); 3) Th2 differentiation: IL4 AND STAT6 AND GATA3 AND JAG1 NOT (INFG AND IL12A AND STAT1 AND TBX21 AND TNF); 4) Treg function: CTLA4 OR JAG1OR (CTLA4 AND JAG1) OR IL10 OR TGFB1 OR (IL10 AND TGFB1) OR (CTLA4 OR JAG1 OR (CTLA4 AND JAG1) AND (IL10 OR TGFB1 OR (IL10 AND TGFB1))); 5) migration: ITGA4 AND (ITGB1 OR ITGB7) (which builds the VLA4 or LPAM adhesion molecule respectively). We then calculated the fragility of the network by using the F coefficient [22], which is defined as the inverse of the average value of the number of interactions participating in a minimal cut set. Robustness is defined as 1–fragility. The five T-cell activation functions described were considered as outputs of the network.

#### 4. Quantitative analysis of the network

In order to measure the weight of each link in the different conditions we calculated the Kullback-Leibler divergence (KL-divergence) for each link of the network using ten groups of samples for each diagnosis, randomly constructed with half of the total samples for each condition. The KL-divergence is a measure that can be used to evaluate the differences between the probability distributions represented by the network, both with the arc corresponding to the relation and without this arc [56].
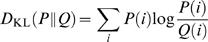



In this case, it is a measure of the mutual information between the parent and the child node in the network. The KL-divergence is a natural distance measure from a “true” probability distribution P to an arbitrary probability distribution Q. Typically P represents data, observations, or a precise calculated probability distribution. The measure Q typically represents a theory, a model, a description or an approximation of P.

### Statistical analysis

The Shapiro-Wilk Test was carried out on each group to assess the normal distribution. Those data groups that were not normal were transformed to base-e-logarithm. We used the *student-*t test to compare individual gene expression levels between MS patients and HC, the influence of IFN-ß treatment in patients with MS, KL-divergence values and gene expression in animal studies. Differences in the clinical course of EAE, histological scores, ELISpot and flow cytometry studies were assessed with the Mann-Whitney U test. The Bonferroni correction for multiple testing was applied as required. For all tests p values of <0.05 were considered as significant. Data was analyzed and processed using the SPSS 13.0 statistical package (SPSS Inc. Chicago, USA).

## Supporting Information

Table S1 (0.11 MB DOC)

Table S2 (0.20 MB DOC)

Table S3 (0.07 MB DOC)

Table S4 (0.08 MB DOC)

Table S5 (0.11 MB DOC)
