# Supplementary material for: A Network Analysis of the Human T-Cell Activation Gene Network Identifies Jagged1 as a Therapeutic Target for Autoimmune Diseases
Source: PLoS One. 2007 Nov 21;2(11):e1222. doi: 10.1371/journal.pone.0001222 (PMC2077806; doi:10.1371/journal.pone.0001222)
Supplement: Table S1 — (0.11 MB DOC) [file pone.0001222.s001.doc]

|  |  |  |
| --- | --- | --- |
|  |  |  |
|  |  |  |
|  |  |  |
|  |  |  |
|  |  |  |
|  |  |  |
|  |  |  |

Table S1: Gene description and PCR assay for the genes in the T-cell activation network (n = 20). Name, gene symbol, Applied TaqMan assay ID and function in the immune system (reference) are shown. References for tables S2 and S3 are provided at the end of the supplementary material.

| Gene Name | **Gene Symbol** | **Assay ID** | **Function** |
| --- | --- | --- | --- |
| *CD28 antigen* | CD28 | Hs00174796_m1 | CD28 costimulation by CD80 and CD86 is essential for CD4-positive T-cell proliferation, survival, interleukin-2 production, and Th2 development (1) |
| *Cytotoxic T-lymphocyte-associated protein 4* | CTLA4 / CD152 | Hs00175480_m1 | CTLA4 is a costimulatory molecule expressed by activated T cells that bind to CD80 and CD86 and transmits an inhibitory signal to T cells (2) |
| *GATA binding protein 3* | GATA3 | Hs00231122_m1 | GATA3 is a transcriptional activator which binds to the enhancer of the T cell receptor alpha and delta genes and controls Th2 cytokine gene expression (3) |
| *Integrin, alpha 4* | ITGA4 / CD49D | Hs00168433_m1 | ITGA4 is expressed on the surface of activated lymphocytes and monocytes and it plays an integral part in their adhesion to the vascular endothelium and migration into the parenchyma (4) |
| *Integrin, beta 1* | ITGB1 / CD29 | Hs00559595_m1 | ITGB1 associates with ITGA4 and mediates the adhesion of monocytes and lymphocytes to the cytokine-activated endothelium (5) |
| *Integrin, beta 7* | ITGB7 | Hs00168469_m1 | ITGB7 associates with ITGA4 and plays a role in the adhesive interactions of leukocytes (6) |
| *Interferon-gamma* | IFNG | Hs00174143_m1 | IFNG is produced by lymphocytes activated by specific antigens or mitogens and, in addition to having antiviral activity, it has important immunoregulatory functions. It is a potent activator of macrophages, it has antiproliferative effects on transformed cells and it can potentate the antiviral and antitumor effects of the type I interferons (7) |
| *Interleukin 10* | IL10 | Hs00174086_m1 | IL10 is a cytokine produced primarily by monocytes and to a lesser extent by lymphocytes that has pleiotropic effects in immunoregulation and inflammation. It down-regulates the expression of Th1 cytokines, including TNF and INFG, MHC class II Ags, and costimulatory molecules on macrophages (8) |
| *Interleukin 12A* | IL12A | Hs00168405_m1 | IL12A is a subunit of a heterodimer cytokine required for the T-cell-independent induction of IFNG, and it is important for the differentiation of both Th1 and Th2 cells (9) |
| *Interleukin 4* | IL4 | Hs00174122_m1 | IL4 is a pleiotropic cytokine produced by activated T cells that participates in at least several B-cell activation processes, as well as those of other cell types. It induces the expression of class II MHC molecules on resting B-cells and enhances both secretion and cell surface expression of IgE and IgG1 (10) |
| *Jagged 1* | JAG1 | Hs00164982_m1 | JAG1 is a NOTCH1 receptor ligand and it directs Th2 differentiation by inducing GATA3 and by directly regulating IL4 gene transcription (11) |
| *Major histocompatibility complex, class II, DQ beta 1* | HLA-DQB1 | Hs00409790_m1 | HLA-DQB1 is a HLA class II beta chain paralogue. It plays a central role in the immune system by presenting peptides derived from extracellular proteins (12) |
| *Major histocompatibility complex, class II, DR alpha* | HLA-DRA | Hs00219575_m1 | HLA-DRA is a HLA class II alpha chain paralogue. It plays a central role in the immune system by presenting peptides derived from extracellular proteins (12) |
| *Myxovirus resistance protein 1* | MX1 / MxA | Hs00182073_m1 | MX1 is inducible by interferons and shows activity against influenza virus and vesicular stomatitis virus (13). Its levels are raised in patients treated with IFN-beta (14) |
| *Protein tyrosine phosphatase, receptor type, C* | PTPRC / CD45 | Hs00365634_g1 | PTPRC is a major high molecular weight leukocyte cell surface molecule that is required for T-cell activation through the antigen receptor (15) |
| *Signal transducer and activator of transcription 1* | STAT1 | Hs00234829_m1 | STAT1 is a transcription factor that in response to IFNG, forms homodimers that are translocate to the nucleus to activate IFNG-responsive genes (16) |
| *Signal transducer and activator of transcription 6* | STAT6 | Hs00598618_m1 | STAT6 is a transcription factor that plays a central role in exerting IL4 mediated biological responses. It carries out a dual function in IL4 signalling: signal transduction and activation of transcription (17) |
| *T-box 21* | TBX21 / T-bet | Hs00203436_m1 | TBX21 is a transcription factor that controls the expression of IFNG. It initiates Th1 lineage development from naive Th precursor cells both by activating Th1 genetic programs and by repressing the opposing Th2 programs (18) |
| *Transforming growth factor, beta 1* | TGFB1 | Hs00171257_m1 | TGFB1 is a multifunctional peptide that controls proliferation, differentiation, and other functions in many cell types. Many cells synthesize TGFB1 and almost all of them have specific receptors for this peptide. Deregulation of TGFB1 activation and signaling may result in apoptosis (19) |
| *Tumor necrosis factor alpha* | TNF | Hs00174128_m1 | TNF is a multifunctional proinflammatory cytokine. It is mainly secreted by macrophages and can induce cell death of certain tumour cell lines. Under certain conditions it can stimulate cell proliferation and induce cell differentiation (20) |
| *Beta-2-microglobulin* | B2M | Hs00187842_m1 | Endogenous control |
| *Glyceraldehyde-3-phosphate dehydrogenase* | GAPDH | Hs99999905_m1 | Endogenous control |

References

1. Andres, P.G., Howland, K.C., Nirula, A., Kane, L.P., Barron, L., Dresnek, D., Sadra, A., Imboden, J., Weiss, A., and Abbas, A.K. 2004. Distinct regions in the CD28 cytoplasmic domain are required for T helper type 2 differentiation. Nat Immunol 5:435-442. Epub 2004 Mar 2007.

2. Magistrelli, G., Jeannin, P., Herbault, N., Benoit De Coignac, A., Gauchat, J.F., Bonnefoy, J.Y., and Delneste, Y. 1999. A soluble form of CTLA-4 generated by alternative splicing is expressed by nonstimulated human T cells. Eur J Immunol 29:3596-3602.

3. Zheng, W., and Flavell, R.A. 1997. The transcription factor GATA-3 is necessary and sufficient for Th2 cytokine gene expression in CD4 T cells. Cell 89:587-596.

4. von Andrian, U.H., and Engelhardt, B. 2003. Alpha4 integrins as therapeutic targets in autoimmune disease. N Engl J Med 348:68-72.

5. Lu, T.T., and Cyster, J.G. 2002. Integrin-mediated long-term B cell retention in the splenic marginal zone. Science 297:409-412.

6. Mora, J.R., Bono, M.R., Manjunath, N., Weninger, W., Cavanagh, L.L., Rosemblatt, M., and Von Andrian, U.H. 2003. Selective imprinting of gut-homing T cells by Peyer's patch dendritic cells. Nature 424:88-93.

7. Pestka, S., Krause, C.D., and Walter, M.R. 2004. Interferons, interferon-like cytokines, and their receptors. Immunol Rev 202:8-32.

8. Kemper, C., Chan, A.C., Green, J.M., Brett, K.A., Murphy, K.M., and Atkinson, J.P. 2003. Activation of human CD4+ cells with CD3 and CD46 induces a T-regulatory cell 1 phenotype. Nature 421:388-392.

9. Liu, Y.J. 2005. IPC: professional type 1 interferon-producing cells and plasmacytoid dendritic cell precursors. Annu Rev Immunol 23:275-306.

10. Stetson, D.B., Voehringer, D., Grogan, J.L., Xu, M., Reinhardt, R.L., Scheu, S., Kelly, B.L., and Locksley, R.M. 2004. Th2 cells: orchestrating barrier immunity. Adv Immunol 83:163-189.

11. Amsen, D., Blander, J.M., Lee, G.R., Tanigaki, K., Honjo, T., and Flavell, R.A. 2004. Instruction of distinct CD4 T helper cell fates by different notch ligands on antigen-presenting cells. Cell 117:515-526.

12. Wake, C.T. 1986. Molecular biology of the HLA class I and class II genes. Mol Biol Med 3:1-11.

13. Haller, O., Frese, M., and Kochs, G. 1998. Mx proteins: mediators of innate resistance to RNA viruses. Rev Sci Tech 17:220-230.

14. Bertolotto, A., Gilli, F., Sala, A., Audano, L., Castello, A., Magliola, U., Melis, F., and Giordana, M.T. 2001. Evaluation of bioavailability of three types of IFNbeta in multiple sclerosis patients by a new quantitative-competitive-PCR method for MxA quantification. J Immunol Methods 256:141-152.

15. Mustelin, T., Vang, T., and Bottini, N. 2005. Protein tyrosine phosphatases and the immune response. Nat Rev Immunol 5:43-57.

16. Darnell, J.E., Jr., Kerr, I.M., and Stark, G.R. 1994. Jak-STAT pathways and transcriptional activation in response to IFNs and other extracellular signaling proteins. Science 264:1415-1421.

17. Quelle, F.W., Shimoda, K., Thierfelder, W., Fischer, C., Kim, A., Ruben, S.M., Cleveland, J.L., Pierce, J.H., Keegan, A.D., Nelms, K., et al. 1995. Cloning of murine Stat6 and human Stat6, Stat proteins that are tyrosine phosphorylated in responses to IL-4 and IL-3 but are not required for mitogenesis. Mol Cell Biol 15:3336-3343.

18. Szabo, S.J., Sullivan, B.M., Stemmann, C., Satoskar, A.R., Sleckman, B.P., and Glimcher, L.H. 2002. Distinct effects of T-bet in TH1 lineage commitment and IFN-gamma production in CD4 and CD8 T cells. Science 295:338-342.

19. Heldin, C.H., Miyazono, K., and ten Dijke, P. 1997. TGF-beta signalling from cell membrane to nucleus through SMAD proteins. Nature 390:465-471.

20. Liu, Z.G. 2005. Molecular mechanism of TNF signaling and beyond. Cell Res 15:24-27.
