# Supplementary material for: A Network Analysis of the Human T-Cell Activation Gene Network Identifies Jagged1 as a Therapeutic Target for Autoimmune Diseases
Source: PLoS One. 2007 Nov 21;2(11):e1222. doi: 10.1371/journal.pone.0001222 (PMC2077806; doi:10.1371/journal.pone.0001222)
Supplement: Table S2 — (0.20 MB DOC) [file pone.0001222.s002.doc]

Table S2: Text mining results of gene interactions of the T-cell activation network. If there is an arc (interaction) from node A to another node B, then we say that A is a parent of B and B is a child of A. In the case that more than one reference is found, we selected the most informative one or a review describing such interactions. References for tables S2 and S3 are provided at the end of the supplementary material.

| **Parent** | **Child** | **Interaction** | **Ref.** |
| --- | --- | --- | --- |
| CD28 | CTLA4 | CD28 increases expression of CTLA4 | (21) |
| CD28 | GATA3 | Activation of GATA3 requires costimulation by CD28 | (22) |
| CD28 | IFNG | CD28 increases expression of IFNG | (23) |
| CD28 | IL10 | Blockade of CD28 costimulation decreases IL 10 | (24) |
| CD28 | IL4 | CD28 increases expression of IL4 | (25) |
| CD28 | PTPRC | CD28 interacts with PTPRC | (26) |
| CD28 | STAT6 | CD28 increases expression of STAT6 | (27) |
| CD28 | TNF | CD28 increases expression of TNF | (23) |
| CTLA4 | STAT6 | CTLA4 decreases STAT6 expression | (28) |
| CTLA4 | TGFB1 | CTLA4 increases expression of TGFB1 | (29) |
| IFNG | HLA-DQB1 | IFNG increases expression of HLA-DQB1 | (30) |
| IFNG | HLA-DRA | IFNG increases expression of HLA-DRA | (31) |
| IFNG | IL10 | IFNG increases expression of IL10 | (32) |
| IFNG | MX1 | IFNG protein increases expression of MX1 gene | (33) |
| IFNG | STAT1 | IFNG increases expression of STAT1 | (34) |
| IFNG | STAT6 | IFNG increases expression of STAT6 | (35) |
| IFNG | TBX21 | IFNG increases expression of TBX21 | (36) |
| IL10 | ITGA4 | IL10 increases expression of ITGA4-ITGB7 complex | (37) |
| IL10 | ITGB7 | IL10 increases expression of ITGA4-ITGB7 complex | (37) |
| IL10 | STAT1 | IL10 decreases expression of STAT1 | (38) |
| IL10 | TNF | IL10 decreases expression of TNF | (39) |
| IL12A | IFNG | IFNG production is increased by IL12 | (32) |
| IL12A | STAT1 | IL12 increases expression of STAT1 | (40) |
| IL4 | IL10 | IL4 increases expression of IL10 | (41) |
| IL4 | IL12A | IL4 decreases expression of IL12 | (42) |
| IL4 | ITGB1 | IL4 increases expression of ITGB1 | (43) |
| IL4 | STAT6 | IL4 induces STAT6 | (44) |
| IL4 | TGFB1 | IL4 increases expression of TGFB1 | (45) |
| ITGA4 | ITGB1 | ITGA4 binds to ITGB1 | (5) |
| ITGA4 | ITGB7 | ITGA4 binds to ITGB7 | (6) |
| JAG1 | GATA3 | JAG1 induces GATA3 | (11) |
| JAG1 | IL4 | JAG1 regulates IL4 expression | (11) |
| JAG1 | TBX21 | JAG1 regulates TBX21 expression | (46) |
| PTPRC | CTLA4 | PTPRC increases expression of CTLA4 | (47) |
| PTPRC | TNF | PTPRC induced expression of TNF protein | (48) |
| STAT1 | GATA3 | STAT1 is required for suppression of GATA3 | (49) |
| STAT1 | STAT6 | STAT1 decreases expression of STAT6 | (50) |
| STAT1 | TBX21 | Activation of STAT1 increases induction of TBX21 | (51) |
| STAT6 | GATA3 | STAT6 increases expression of GATA3 | (52) |
| TBX21 | GATA3 | TBX21 interacts with GATA3 | (53) |
| TGFB1 | GATA3 | TGFB1 decreases expression of GATA3 | (54) |
| TGFB1 | IFNG | TGFB1 decreases the production of IFNG | (55) |
| TGFB1 | IL10 | TGFB1 increases expression of IL10 | (56) |
| TGFB1 | ITGA4 | TGFB1 increases expression of ITGA4 | (57) |
| TGFB1 | ITGB1 | TGFB1 increases expression of ITGB1 | (58) |
| TGFB1 | ITGB7 | TGFB1 increases expression of the ITGB7-ITGAE complex | (59) |
| TGFB1 | TBX21 | TGFB1 decreases expression of TBX21 | (60) |
| TNF | ITGA4 | TNF increases expression of ITGA4 | (61) |
| TNF | ITGB7 | TNF increases expression of ITGB7 | (61) |
| TNF | MX1 | TNF increases expression of MX1 | (62) |

References

21. Tsai, M.K., Ho, H.N., Chien, H.F., Tzeng, M.C., Chen, C.H., and Lee, P.H. 2005. Multiple negative feedbacks on CD152 expression in allograft tolerance. Transplantation 79:174-181.

22. Rodriguez-Palmero, M., Hara, T., Thumbs, A., and Hunig, T. 1999. Triggering of T cell proliferation through CD28 induces GATA-3 and promotes T helper type 2 differentiation in vitro and in vivo. Eur J Immunol 29:3914-3924.

23. Rao, A., Luo, C., and Hogan, P.G. 1997. Transcription factors of the NFAT family: regulation and function. Annu Rev Immunol 15:707-747.

24. Favali, C., Costa, D., Afonso, L., Conceicao, V., Rosato, A., Oliveira, F., Costa, J., Barral, A., Barral-Netto, M., and Brodskyn, C.I. 2005. Role of costimulatory molecules in immune response of patients with cutaneous leishmaniasis. Microbes Infect 7:86-92. Epub 2004 Dec 2013.

25. Skapenko, A., Lipsky, P.E., Kraetsch, H.G., Kalden, J.R., and Schulze-Koops, H. 2001. Antigen-independent Th2 cell differentiation by stimulation of CD28: regulation via IL-4 gene expression and mitogen-activated protein kinase activation. J Immunol 166:4283-4292.

26. Zocchi, M.R., Poggi, A., Crosti, F., Tongiani, S., and Rugarli, C. 1992. Signalling in human tumour infiltrating lymphocytes: the CD28 molecule is functional and is physically associated with the CD45R0 molecule. Eur J Cancer 28A:749-754.

27. Oki, S., Otsuki, N., Kohsaka, T., and Azuma, M. 2000. Stat6 activation and Th2 cell differentiation [correction of proliferation] driven by CD28 [correction of CD28 signals]. Eur J Immunol 30:1416-1424.

28. Finotto, S., and Glimcher, L. 2004. T cell directives for transcriptional regulation in asthma. Springer Semin Immunopathol 25:281-294. Epub 2003 Nov 2012.

29. Schneider, H., Mandelbrot, D.A., Greenwald, R.J., Ng, F., Lechler, R., Sharpe, A.H., and Rudd, C.E. 2002. Cutting edge: CTLA-4 (CD152) differentially regulates mitogen-activated protein kinases (extracellular signal-regulated kinase and c-Jun N-terminal kinase) in CD4+ T cells from receptor/ligand-deficient mice. J Immunol 169:3475-3479.

30. Gonalons, E., Barrachina, M., Garcia-Sanz, J.A., and Celada, A. 1998. Translational control of MHC class II I-A molecules by IFN-gamma. J Immunol 161:1837-1843.

31. Rohn, W., Tang, L.P., Dong, Y., and Benveniste, E.N. 1999. IL-1 beta inhibits IFN-gamma-induced class II MHC expression by suppressing transcription of the class II transactivator gene. J Immunol 162:886-896.

32. Rossi, D., and Zlotnik, A. 2000. The biology of chemokines and their receptors. Annu Rev Immunol 18:217-242.

33. Andrews, H.N., Mullan, P.B., McWilliams, S., Sebelova, S., Quinn, J.E., Gilmore, P.M., McCabe, N., Pace, A., Koller, B., Johnston, P.G., et al. 2002. BRCA1 regulates the interferon gamma-mediated apoptotic response. J Biol Chem 277:26225-26232. Epub 22002 May 26213.

34. Hu, X., Herrero, C., Li, W.P., Antoniv, T.T., Falck-Pedersen, E., Koch, A.E., Woods, J.M., Haines, G.K., and Ivashkiv, L.B. 2002. Sensitization of IFN-gamma Jak-STAT signaling during macrophage activation. Nat Immunol 3:859-866. Epub 2002 Aug 2012.

35. Frucht, D.M., Aringer, M., Galon, J., Danning, C., Brown, M., Fan, S., Centola, M., Wu, C.Y., Yamada, N., El Gabalawy, H., et al. 2000. Stat4 is expressed in activated peripheral blood monocytes, dendritic cells, and macrophages at sites of Th1-mediated inflammation. J Immunol 164:4659-4664.

36. Lighvani, A.A., Frucht, D.M., Jankovic, D., Yamane, H., Aliberti, J., Hissong, B.D., Nguyen, B.V., Gadina, M., Sher, A., Paul, W.E., et al. 2001. T-bet is rapidly induced by interferon-gamma in lymphoid and myeloid cells. Proc Natl Acad Sci U S A 98:15137-15142.

37. Roy, M.P., Kim, C.H., and Butcher, E.C. 2002. Cytokine control of memory B cell homing machinery. J Immunol 169:1676-1682.

38. Shen, X., Hong, F., Nguyen, V.A., and Gao, B. 2000. IL-10 attenuates IFN-alpha-activated STAT1 in the liver: involvement of SOCS2 and SOCS3. FEBS Lett 480:132-136.

39. Cassatella, M.A., Meda, L., Bonora, S., Ceska, M., and Constantin, G. 1993. Interleukin 10 (IL-10) inhibits the release of proinflammatory cytokines from human polymorphonuclear leukocytes. Evidence for an autocrine role of tumor necrosis factor and IL-1 beta in mediating the production of IL-8 triggered by lipopolysaccharide. J Exp Med 178:2207-2211.

40. Maier, J., Kincaid, C., Pagenstecher, A., and Campbell, I.L. 2002. Regulation of signal transducer and activator of transcription and suppressor of cytokine-signaling gene expression in the brain of mice with astrocyte-targeted production of interleukin-12 or experimental autoimmune encephalomyelitis. Am J Pathol 160:271-288.

41. Nelms, K., Keegan, A.D., Zamorano, J., Ryan, J.J., and Paul, W.E. 1999. The IL-4 receptor: signaling mechanisms and biologic functions. Annu Rev Immunol 17:701-738.

42. Lubberts, E., Joosten, L.A., Chabaud, M., van Den Bersselaar, L., Oppers, B., Coenen-De Roo, C.J., Richards, C.D., Miossec, P., and van Den Berg, W.B. 2000. IL-4 gene therapy for collagen arthritis suppresses synovial IL-17 and osteoprotegerin ligand and prevents bone erosion. J Clin Invest 105:1697-1710.

43. Jinquan, T., Quan, S., Feili, G., Larsen, C.G., and Thestrup-Pedersen, K. 1999. Eotaxin activates T cells to chemotaxis and adhesion only if induced to express CCR3 by IL-2 together with IL-4. J Immunol 162:4285-4292.

44. Schaffer, A., Cerutti, A., Shah, S., Zan, H., and Casali, P. 1999. The evolutionarily conserved sequence upstream of the human Ig heavy chain S gamma 3 region is an inducible promoter: synergistic activation by CD40 ligand and IL-4 via cooperative NF-kappa B and STAT-6 binding sites. J Immunol 162:5327-5336.

45. Kohyama, M., Sugahara, D., Hosokawa, H., Kubo, M., and Hozumi, N. 2001. IL-4-mediated development of TGF-beta1-producing cells from naive CD4(+) T cells through a STAT6-independent mechanism. Eur J Immunol 31:3659-3666.

46. Minter, L.M., Turley, D.M., Das, P., Shin, H.M., Joshi, I., Lawlor, R.G., Cho, O.H., Palaga, T., Gottipati, S., Telfer, J.C., et al. 2005. Inhibitors of gamma-secretase block in vivo and in vitro T helper type 1 polarization by preventing Notch upregulation of Tbx21. Nat Immunol 6:680-688.

47. Fecteau, S., Basadonna, G.P., Freitas, A., Ariyan, C., Sayegh, M.H., and Rothstein, D.M. 2001. CTLA-4 up-regulation plays a role in tolerance mediated by CD45. Nat Immunol 2:58-63.

48. Hayes, A.L., Smith, C., Foxwell, B.M., and Brennan, F.M. 1999. CD45-induced tumor necrosis factor alpha production in monocytes is phosphatidylinositol 3-kinase-dependent and nuclear factor-kappaB-independent. J Biol Chem 274:33455-33461.

49. Lucas, S., Ghilardi, N., Li, J., and de Sauvage, F.J. 2003. IL-27 regulates IL-12 responsiveness of naive CD4+ T cells through Stat1-dependent and -independent mechanisms. Proc Natl Acad Sci U S A 100:15047-15052. Epub 12003 Dec 15041.

50. Yu, C.R., Mahdi, R.M., Ebong, S., Vistica, B.P., Chen, J., Guo, Y., Gery, I., and Egwuagu, C.E. 2004. Cell proliferation and STAT6 pathways are negatively regulated in T cells by STAT1 and suppressors of cytokine signaling. J Immunol 173:737-746.

51. Villarino, A.V., Huang, E., and Hunter, C.A. 2004. Understanding the pro- and anti-inflammatory properties of IL-27. J Immunol 173:715-720.

52. Zhou, M., and Ouyang, W. 2003. The function role of GATA-3 in Th1 and Th2 differentiation. Immunol Res 28:25-37.

53. Hwang, E.S., Szabo, S.J., Schwartzberg, P.L., and Glimcher, L.H. 2005. T helper cell fate specified by kinase-mediated interaction of T-bet with GATA-3. Science 307:430-433.

54. Heath, V.L., Murphy, E.E., Crain, C., Tomlinson, M.G., and O'Garra, A. 2000. TGF-beta1 down-regulates Th2 development and results in decreased IL-4-induced STAT6 activation and GATA-3 expression. Eur J Immunol 30:2639-2649.

55. Sudarshan, C., Galon, J., Zhou, Y., and O'Shea, J.J. 1999. TGF-beta does not inhibit IL-12- and IL-2-induced activation of Janus kinases and STATs. J Immunol 162:2974-2981.

56. Wang, S.C., Ohata, M., Schrum, L., Rippe, R.A., and Tsukamoto, H. 1998. Expression of interleukin-10 by in vitro and in vivo activated hepatic stellate cells. J Biol Chem 273:302-308.

57. Ni, J., Chen, S.F., and Hollander, D. 1996. Effects of dextran sulphate sodium on intestinal epithelial cells and intestinal lymphocytes. Gut 39:234-241.

58. Wahl, S.M., Allen, J.B., Weeks, B.S., Wong, H.L., and Klotman, P.E. 1993. Transforming growth factor beta enhances integrin expression and type IV collagenase secretion in human monocytes. Proc Natl Acad Sci U S A 90:4577-4581.

59. Parker, C.M., Cepek, K.L., Russell, G.J., Shaw, S.K., Posnett, D.N., Schwarting, R., and Brenner, M.B. 1992. A family of beta 7 integrins on human mucosal lymphocytes. Proc Natl Acad Sci U S A 89:1924-1928.

60. Gorelik, L., Constant, S., and Flavell, R.A. 2002. Mechanism of transforming growth factor beta-induced inhibition of T helper type 1 differentiation. J Exp Med 195:1499-1505.

61. Puig-Kroger, A., Sanz-Rodriguez, F., Longo, N., Sanchez-Mateos, P., Botella, L., Teixido, J., Bernabeu, C., and Corbi, A.L. 2000. Maturation-dependent expression and function of the CD49d integrin on monocyte-derived human dendritic cells. J Immunol 165:4338-4345.

62. Cui, K., Tailor, P., Liu, H., Chen, X., Ozato, K., and Zhao, K. 2004. The chromatin-remodeling BAF complex mediates cellular antiviral activities by promoter priming. Mol Cell Biol 24:4476-4486.

63. Tsukumo, S., and Yasutomo, K. 2004. Notch governing mature T cell differentiation. J Immunol 173:7109-7113.
