# Supplementary material for: A Network Analysis of the Human T-Cell Activation Gene Network Identifies Jagged1 as a Therapeutic Target for Autoimmune Diseases
Source: PLoS One. 2007 Nov 21;2(11):e1222. doi: 10.1371/journal.pone.0001222 (PMC2077806; doi:10.1371/journal.pone.0001222)
Supplement: Table S3 — (0.07 MB DOC) [file pone.0001222.s003.doc]

Table S3: Single gene expression levels and differences between HC and MS patients, untreated or treated with IFN-ß: We compared the differences in the gene expression levels between MS patients and HC at the single gene expression level. We found no significant difference in the gene expression levels between MS and HC after correcting for multiple testing. We found that IFN-ß treated patients had a significant increase in the MX1 gene expression levels (p=0.031) compared to untreated patients, because MX1 is a well known marker of IFN-ß bioavailability (14), validating our experimental method as well as our sample handling and processing. The results are described by median (rank) and p values were adjusted using the Bonferroni method.

|  | **HC**  **N = 52** | **MS (untreated)**  **N = 27** | **MS IFN-ß**  **N = 25** | **p value** |
| --- | --- | --- | --- | --- |
| CD28 | 2.49E+01  (1.05E+00-1.34E+02) | 1.30E+01  (2.95E-01-1.04E+02) | 3.73E+01  (7.31E+00-1.06E+02) | ns |
| CTLA4 | 3.64E-02  (1.89E-03-1.58E+00) | 5.70E-02  (8.99E-04-7.65E-01) | 5.62E-02  (2.91E-03-7.47E-01) | ns |
| GATA3 | 2.35E+02  (1.81E+01-1.70E+03) | 1.85E+02  (1.47E+01-3.10E+03) | 2.93E+02  (3.75E+01-2.46E+03) | ns |
| ITGA4 | 1.49E+03  (1.33E+02-1.21E+04) | 1.29E+03  (2.32E+02-1.16E+04) | 1.98E+03  (4.56E+02-1.21E+04) | ns |
| ITGB1 | 7.01E+02  (7.70E+01-3.98E+03) | 5.32E+02  (1.61E+02-3.18E+03) | 8.77E+02  (2.67E+02-2.44E+03) | ns |
| ITGB7 | 1.03E+01  (1.10E+00-6.21E+01) | 5.91E+00  (2.05E+00-1.17E+02) | 9.99E+00  (3.27E+00-4.31E+01) | ns |
| IFNG | 4.77E-01  (3.40E-02-4.89E+00) | 2.28E-01  (2.21E-02-1.13E+00) | 4.43E-01  (2.93E-02-2.39E+00) | ns |
| IL10 | 1.23E-03  (9.69E-05-3.19E-02) | 1.69E-03  (2.66E-04-3.19E-02) | 4.02E-03  (1.11E-05-2.59E-02) | ns |
| IL12A | 3.91E+00  (7.05E-02-5.02E+01) | 4.60E+00  (7.20E-01-2.65E+01) | 1.01E+01  (1.95E-02-4.04E+01) | ns |
| IL4 | 1.85E-01  (4.56E-03-8.21E+00) | 8.13E-02  (1.29E-02-7.08E-01) | 2.40E-01  (3.48E-02-9.81E-01) | ns |
| JAG1 | 3.92E-01  (4.04E-02-2.96E+00) | 2.87E-01  (4.51E-02-3.23E+00) | 3.12E-01  (5.43E-02-1.49E+00) | ns |
| HLA-DQB1 | 1.67E+01  (4.06E+00-8.68E+01) | 1.69E+01  (1.33E+00-7.28E+01) | 1.59E+01  (6.68E+00-5.79E+01) | ns |
| HLA-DRA | 2.17E+02  (6.03E+01-6.79E+02) | 1.90E+02  (2.43E+01-5.26E+02) | 2.32E+02  (1.03E+02-5.91E+02) | ns |
| MX1 | 2.09E+01  (1.51E+00-4.23E+02) | 2.22E+01  (4.15E+00-2.32E+02) | 8.37E+01  (7.94E+00-1.06E+03) | 0.031^*^ |
| PTPRC | 1.37E+00  (7.02E-02-1.16E+01) | 8.32E-01  (1.91E-01-4.83E+00) | 1.64E+00  (1.46E-01-6.41E+00) | ns |
| STAT1 | 7.15E+01  (7.42E+00-6.62E+02) | 8.55E+01  (1.22E+01-8.69E+02) | 1.70E+02  (1.76E+01-8.01E+02) | ns |
| STAT6 | 2.76E+03  (1.80E+02-1.54E+04) | 1.84E+03  (3.52E+02-1.17E+04) | 4.92E+03  (1.09E+03-2.35E+04) | ns |
| TBX21 | 2.38E+01  (1.35E+00-2.79E+02) | 1.23E+01  (4.97E-01-1.12E+02) | 1.95E+01  (1.66E+00-1.44E+02) | ns |
| TGFB1 | 1.72E+03  (2.26E+02-9.53E+03) | 1.44E+03  (4.02E+02-6.22E+03) | 2.70E+03  (4.97E+02-9.04E+03) | ns |
| TNF | 8.17E-02  (5.19E-03-1.27E+00) | 5.15E-02  (1.15E-02-4.31E-01) | 6.50E-02  (1.99E-02-3.91E-01) | ns |

# ns = not significant

# *p value between untreated and IFN-beta treated patients
