# Supplementary material for: A Network Analysis of the Human T-Cell Activation Gene Network Identifies Jagged1 as a Therapeutic Target for Autoimmune Diseases
Source: PLoS One. 2007 Nov 21;2(11):e1222. doi: 10.1371/journal.pone.0001222 (PMC2077806; doi:10.1371/journal.pone.0001222)
Supplement: Table S4 — (0.08 MB DOC) [file pone.0001222.s004.doc]

Table S4: Single gene expression levels from *in vitro* assays using PBMCs from untreated MS patients and those stimulated with Jagged 1, IFN-ß or Jagged 1 + IFN-ß: the levels of the HES5 gene were used as reporter of Jagged 1-Notch activation (63) and the levels of MX1 were used as a reporter of IFN-B bioavailability (14). The results are presented as medians (rank) and the p values were adjusted using the Bonferroni method.

|  | **Baseline** | **Jagged 1** | **IFN-ß** | **Jagged 1 + IFN-ß** | **p value** |
| --- | --- | --- | --- | --- | --- |
| CD28 | 1.01E+02  (4.12E-01-1.28E+03) | 1.48E+01  (1.70E-01-2.02E+02) | 2.20E+01  (1.25E-01-3.29E+02) | 4.51E+02  (6.08E-02-1.07E+04) | ns |
| CTLA4 | 1.74E+01  (1.23E-01-1.73E+02) | 1.74E+00  (5.32E-02-1.10E+01) | 8.52E+00  (9.04E-02-1.21E+02) | 1.68E+01  (8.42E-02-3.69E+02) | ns |
| GATA3 | 2.95E+02  (8.03E-01-2.64E+03) | 6.19E+01  (7.35E-01-7.40E+02) | 5.97E+01  (3.30E-01-5.56E+02) | 1.77E+03  (4.21E-01-4.18E+04) | ns |
| HES5 | 9.98E-07  (3.40E-07-2.05E-06) | 1.20E-05  (1.82E-06-3.75E-05) | 8.78E-07  (5.33E-07-1.99E-06) | 2.38E-05  (4.21E-06-7.26E-05) | 0.028^a^ 0.034^c^ |
| HLA-DQB1 | 7.49E+02  (2.07E+00-1.23E+04) | 2.48E+01  (1.47E+00-1.45E+02) | 7.80E+01  (9.30E-01-1.07E+03) | 4.40E+02  (7.90E-01-1.01E+04) | ns |
| HLA-DRA | 2.65E+03  (1.58E+01-2.31E+04) | 2.45E+02  (1.10E+01-2.66E+03) | 1.20E+03  (4.02E+00-2.19E+04) | 5.09E+03  (3.86E+00-1.18E+05) | ns |
| IFNG | 1.63E+01  (3.51E-02-1.86E+02) | 1.16E+00  (6.32E-03-1.04E+01) | 6.34E+00  (7.47E-03-1.11E+02) | 6.30E+01  (1.82E-03-1.49E+03) | ns |
| IL10 | 4.70E-01  (6.64E-03-3.07E+00) | 2.43E-01  (1.04E-02-2.19E+00) | 3.33E+00  (1.43E-02-6.07E+01) | 9.08E+00  (5.16E-03-2.02E+02) | ns |
| IL12A | 1.11E+01  (3.03E-02-1.58E+02) | 2.98E-01  (1.33E-02-3.56E+00) | 1.87E+00  (2.59E-03-3.76E+01) | 2.06E+01  (3.24E-03-4.89E+02) | ns |
| IL4 | 1.13E+00  (1.02E-03-1.74E+01) | 4.52E-02  (2.03E-04-4.95E-01) | 2.03E-01  (5.54E-04-2.67E+00) | 3.75E+00  (2.35E-04-8.88E+01) | ns |
| ITGA4 | 4.91E+03  (1.14E+01-7.87E+04) | 1.12E+02  (2.28E+00-1.27E+03) | 3.81E+02  (7.45E-01-5.64E+03) | 4.64E+03  (9.86E-01-1.10E+05) | ns |
| ITGB1 | 5.11E+03  (6.15E+00-5.24E+04) | 5.18E+02  (3.00E+00-7.51E+03) | 4.23E+02  (8.91E-01-7.36E+03) | 1.70E+04  (1.11E+00-4.06E+05) | ns |
| ITGB7 | 1.97E+03  (1.51E+00-3.91E+04) | 4.38E+01  (4.31E-01-4.98E+02) | 5.08E+02  (6.74E-01-1.05E+04) | 3.83E+03  (7.89E-01-9.13E+04) | ns |
| JAG1 | 1.00E+02  (1.74E-01-9.33E+02) | 1.95E+01  (1.88E-01-2.38E+02) | 3.87E+01  (9.62E-02-6.73E+02) | 1.25E+03  (5.76E-02-2.97E+04) | ns |
| MX1 | 4.72E+01  (7.63E-01-2.66E+02) | 4.50E+02  (4.44E-01-7.34E+03) | 9.15E+02  (4.58E+00-1.41E+04) | 1.58E+04  (1.60E+00-3.72E+05) | 0.038^b^ 0.014^c^ |
| PTPRC | 1.65E+04  (3.03E+01-2.71E+05) | 5.88E+02  (1.70E+01-4.33E+03) | 3.59E+03  (1.10E+01-6.62E+04) | 3.62E+04  (1.68E+01-8.60E+05) | ns |
| STAT1 | 2.01E+02  (2.85E+00-1.16E+03) | 3.97E+01  (2.59E+00-2.90E+02) | 2.99E+02  (2.47E+00-4.37E+03) | 1.57E+03  (2.65E+00-3.53E+04) | ns |
| STAT6 | 3.30E+02  (2.78E+00-4.56E+03) | 6.69E+01  (9.50E-01-5.75E+02) | 6.74E+01  (6.66E-01-7.10E+02) | 1.07E+03  (8.76E-01-2.45E+04) | ns |
| TBX21 | 1.48E+03  (1.59E+00-2.23E+04) | 1.21E+02  (7.67E-01-1.64E+03) | 2.89E+02  (3.14E-01-5.29E+03) | 5.85E+03  (2.36E-01-1.39E+05) | ns |
| TGFB1 | 3.80E+04  (2.24E+01-7.60E+05) | 9.09E+02  (7.90E+00-1.24E+04) | 3.60E+03  (6.07E+00-7.14E+04) | 1.43E+05  (4.40E+00-3.42E+06) | ns |
| TNF | 6.93E+00  (1.71E-01-4.64E+01) | 9.40E-01  (5.83E-02-3.22E+00) | 4.15E+00  (8.67E-02-5.58E+01) | 1.04E+01  (2.77E-02-2.15E+02) | ns |

# ns = not significant

^a^p value between basal and Jagged 1

^b^p value between basal and IFN-beta

^c^p value between basal and Jagged1 + IFN-beta
