# Supplementary material for: A Network Analysis of the Human T-Cell Activation Gene Network Identifies Jagged1 as a Therapeutic Target for Autoimmune Diseases
Source: PLoS One. 2007 Nov 21;2(11):e1222. doi: 10.1371/journal.pone.0001222 (PMC2077806; doi:10.1371/journal.pone.0001222)
Supplement: Table S5 — (0.11 MB DOC) [file pone.0001222.s005.doc]

**Table S5.** Gene expression studies in splenocytes from EAE animals untreated or with EAE treated or untreated with Jagged1 peptide. Results are described as median (rank).

|  | **Day 9** | | | **day 30** | | |
| --- | --- | --- | --- | --- | --- | --- |
| **Gene** | **Control** | **JAG1** | **p value** | **Control** | **JAG1** | **p value** |
| CD25 | 2.20E-03  (7.97E-04-6.33E-03) | 3.01E-03  (1.77E-03-4.02E-03) | ns | 8.86E-04  (8.43E-04-9.29E-04) | 5.93E-04  (5.14E-04-6.71E-04) | ns |
| Ctla4 | 1.81E-03  (9.24E-04-4.08E-03) | 2.97E-03  (1.03E-03-1.48E-02) | ns | 6.47E-04  (6.13E-04-6.81E-04) | 9.63E-04  (7.88E-04-1.14E-03) | ns |
| Foxp3 | 2.80E-03  (8.08E-04-7.33E-03) | 2.15E-03  (1.91E-03-6.46E-03) | ns | 1.06E-03  (5.10E-04-1.61E-03) | 2.81E-03  (2.46E-03-3.16E-03) | ns |
| Il10 | 7.20E-03  (1.41E-03-1.19E-02) | 5.48E-03  (1.66E-03-1.33E-02) | 0.029 | 2.91E-03  (1.78E-03-4.03E-03) | 1.43E-03  (5.23E-04-3.38E-03) | ns |
| Il17a | 1.07E-03  (7.76E-04-1.55E-03) | 9.30E-03  (6.52E-03-3.04E-02) | 0.028 | 6.13E-04  (5.93E-04-6.34E-04) | 4.01E-07  (3.63E-07-4.38E-07) | ns |
| Il4 | 3.07E-03  (1.77E-03-8.25E-03) | 1.09E-03  (8.83E-04-1.44E-03) | ns | 4.37E-07  (4.15E-07-4.60E-07) | 1.81E-07  (1.62E-07-1.99E-07) | ns |
| Indo | 5.16-07  (8.42E-07-2.07E-06) | 8.21E-08  (6.49E-10-4.65E-08) | ns | 9.15E-07  (7.98E-07-1.03E-06) | 1.92E-08  (3.40E-10-3.88E-08) | ns |
| Ifng | 1.18E-04  (1.07E-04-1.29E-04) | 6.55E-04  (2.93E-04-1.36E-03) | ns | 2.95E-03  (2.65E-03-3.25E-03) | 6.78E-04  (5.72E-04-7.84E-04) | ns |
| Itga4 | 4.15E-01  (1.59E-01-5.05E-01) | 3.20E-01  (2.86E-01-3.63E-01) | ns | 1.09E-01  (6.63E-02-2.84E-01) | 6.02E-02  (5.79E-02-6.24E-02) | ns |
| Itgb1 | 7.77E-02  (3.99E-02-8.50E-02) | 6.96E-02  (6.39E-02-7.40E-02) | ns | 1.06E-01  (6.43E-02-1.47E-01) | 5.78E-02  (5.54E-02-6.03E-02) | ns |
| Itgb7 | 2.31E-01  (9.38E-02-3.54E-01) | 1.05E-01  (5.64E-02-2.51E-01) | ns | 1.14E-01  (4.67E-02-1.82E-01) | 2.92E-02  (1.58E-02-4.27E-02) | 0.012 |
| Rorc | 3.19E-03  (1.72E-03-5.90E-03) | 1.89E-03  (8.06E-04-2.44E-03) | ns | 3.64E-04  (3.24E-04-4.03E-04) | 4.33E-07  (3.88E-07-4.78E-07) | ns |
| Tgfb1 | 1.70E-01  (1.06E-01-2.62E-01) | 1.00E-01  (6.38E-02-1.88E-01) | ns | 1.45E-01  (1.15E-01-1.74E-01) | 4.84E-02  (3.89E-02-5.79E-02) | 0.010 |
| Tnf | 6.74E-02  (2.50E-02-1.37E-01) | 1.79E-02  (1.35E-02-2.03E-02) | 0.032 | 3.08E-02  (1.94E-02-4.21E-02) | 8.45E-03  (7.12E-03-9.79E-03) | 0.010 |
